# Beyond Traditional Lateral Flow Assays: Enhancing Performance Through Multianalytical Strategies

**DOI:** 10.3390/bios15020068

**Published:** 2025-01-23

**Authors:** Eleni Lamprou, Panagiota M. Kalligosfyri, Despina P. Kalogianni

**Affiliations:** Department of Chemistry, University of Patras, Rio, GR26504 Patras, Greece; chem3099@upnet.gr

**Keywords:** strip, rapid test, nanoparticles, immunoassay, nucleic acids, visual detection, biosensor, sensor, multiplex, point-of-care testing

## Abstract

Multiplex lateral flow assays are one of the greatest advancements in the world of rapid diagnostics, achieving the performance of several tests in one. These tests meet the basic requirements of increasing ease of use, low detection limit, and high specificity, as they combine the use of novel strategies, such as the exploitation of multiple detection labels, and a variety of amplification methods. These tests have proven their usefulness in many different areas, including clinical diagnostics, food, and environmental monitoring. In this review paper, we attempt to highlight and discuss the predominant changes in multianalyte LFAs, as related to their principle, their development, and their combination with other methods. Attention is paid to their flexibility and the challenges associated with the use of LFA arrays, including strategies to improve the detectability, sensitivity, and reliability of the assays. Therefore, this review emphasizes the current advances in the field to underline the possible impact of multiplex LFAs on the future of diagnostics and analytical sciences.

## 1. Introduction

Lateral flow assays (LFAs) have become a cornerstone in diagnostic testing, particularly in point-of-care (POC) applications, due to their inherent advantages of simplicity, rapidity, portability, and cost-effectiveness. They can detect several targets in a few minutes, without the need for highly trained personnel or expensive and sophisticated instrumentation. These paper-based devices leverage capillary action to facilitate the movement of liquid samples through a membrane, where specific reagents interact with target analytes to produce detectable and visual signals. The accessibility, ease of use, and operation of LFAs have led to widespread adoption in various fields, including medical diagnostics, environmental monitoring, and food safety [1,2,3]. The great achievements of nanotechnology have boosted the efficacy of LFAs. LFAs have widely exploited the use of nanoparticles and other nanomaterials, which enhanced the signal output and the overall analytical performance of LFAs due to their unique optical properties [4]. However, traditional LFAs are often limited to the detection of single analytes, which restricts their utility in complex sample matrices, where multiple biomarkers may be present simultaneously [3].

Recent advancements in multiplex lateral flow assays now aim at addressing these limitations by allowing the detection of multiple targets in one sample. The multiplexing capability not only improves diagnostic precision but also enhances efficiency by reducing the sample volume and reagents required for testing. Innovations such as micro-dispensing technologies and improved diagnostic membrane architectures are being explored to boost sensitivity and specificity, thereby overcoming challenges associated with traditional LFAs [5]. Furthermore, integrating advanced detection methods, like surface-enhanced Raman scattering (SERS) and fluorescence, has shown significant promise in increasing the detectability of LFAs, allowing for the detection of low-abundance biomarkers [6].

The rapid evolution of multianalytical LFAs is also driven by the increasing demand for rapid and reliable diagnostics in response to global health challenges, such as infectious disease outbreaks and chronic disease management. The COVID-19 pandemic underlined the urgent need for efficient testing solutions that can be deployed swiftly in various settings. As a result, researchers are focusing on enhancing the performance characteristics of LFAs through novel materials and innovative assay designs that can accommodate diverse applications—from detecting pathogens to monitoring therapeutic drug levels [3]. This review aims to provide a comprehensive overview of the current state of multianalytical LFAs, highlighting recent technological advancements, practical applications, and future research directions in this rapidly evolving field.

## 2. Multiplex Lateral Flow Immunoassays

Lateral flow immunoassays are widely adopted in diagnostics as rapid antigen detection enables mass population monitoring or mass sample analysis and can be applied for individual analysis. The LFA principle typically involves the immobilization of biorecognition molecules on a diagnostic membrane, usually a nitrocellulose membrane, targeting different targets, while the detection is usually visual by using colorimetric, fluorescent, or chemiluminescent reporters. LFAs consist of four distinct parts: an immersion pad that is inserted into a proper developing solution; a sample pad or conjugation pad, where the conjugated reporters (nanoparticles) and the sample are added; the diagnostic membrane; and an absorption pad to ensure continuous flow across the lateral flow strip. All pads are properly assembled with overlapping ends onto an adhesive backing card. Several recognition biomolecules, such as proteins, antibodies, and DNA oligonucleotides, are immobilized at specific areas of the diagnostic membrane to construct the test and the control lines of the strip. Immobilization of the molecules is accomplished by drying the membrane at room temperature, in an oven at a higher temperature, or under UV irradiation. The conjugates applied on the conjugate pad are also dried; therefore, a dry-reagent strip is prepared. Upon the addition of the sample, the strip is immersed into a suitable developing solution that moves upward due to capillary action, re-hydrating and drifting all the reagents through the strip. The developing solution contains all the necessary surfactants for increased detectability and specificity of the test. The analytes are then captured specifically at the test zones of the strip through interaction with the immobilized molecules and detected by the accumulation of the reporters, forming visual colored or fluorescent lines. A last visual line is also formed at the control zone of the strip to ensure that the test is valid. Multiplexing analysis is the current aim of biomolecular analysis, in order to detect multiple analytes in one sample and obtain more information.

Depending on the approach, assay principle, number of target analytes, and detection method, various multiple labeling strategies are employed to enhance sensitivity and specificity. These strategies include the use of nanomaterials such as gold nanoparticles, nano-urchins, and silver nanoparticles, which are conjugated with specific antibodies corresponding to the targets. SERS probes, such as core-shell particles (e.g., Au-Ag), are also widely used and further conjugated with specific antibodies. Additionally, specific DNA probes labeled with proteins (e.g., streptavidin), small molecules (e.g., biotin), and fluorescent dyes with non-overlapping spectra are frequently utilized for multiplexing. Nanozymes are novel molecules used for signal enhancement. Signal amplification was also accomplished using hybridization chain reaction or catalytic hairpin assembly. Other approaches involve target amplification through recombinase polymerase isothermal amplification (RPA), polymerase chain reaction (PCR), or other isothermal amplification techniques to identify and specifically amplify target sequences. After amplification, the detection of these products occurs using the aforementioned strategies, including nanoparticle–antibody conjugates, fluorescent dyes, or protein labels. Some methods combine these techniques, such as detecting PCR products that have been specifically amplified and subsequently labeled with multiple specific tags. Hybrid approaches, such as combining non-fluorescent (e.g., for visual detection) and fluorescent labels, further enhance detection capabilities, making these systems versatile and highly effective for diverse analytical applications. Finally, clustered regularly interspaced short palindromic repeats system (CRISPR) in combination with CRISPR-associated (Cas) enzymes have been exploited for signal amplification. Representative examples and their detection mechanisms are thoroughly described and categorized in the following sections.

**Figure 1:**
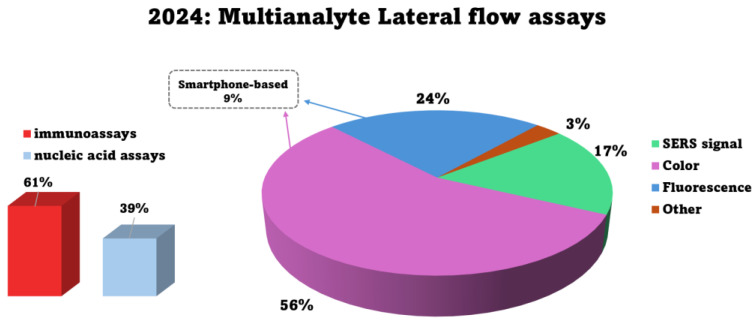
An overview of the reports related to assay principles.

In brief summary, the thorough reviewing of the recently reported multianalyte, i.e., multiplex, LFAs revealed that 61% are based on immunoassay principles, utilizing specific conjugates of corresponding antibodies with nanoparticles and specific antibodies that were used to form the test lines of the lateral flow strip, while 39% employ nucleic acid-based approaches such as PCR, primer extension reaction (PEXT), RPA, etc. Among all these LFAs, 56% report colorimetric detection—most commonly employing gold nanoparticle (AuNP) conjugates—with results visible to the naked eye. Additionally, 24% utilize fluorescence-based detection, and 17% are based on the SERS signal. Smartphone integration is featured in 9% of the assays, facilitating not only data sharing and qualitative analysis but also quantitative interpretation through the use of designated applications for target quantification (Figure 1).

### 2.1. Simple Colorimetric Lateral Flow Immunoassays

**Figure 2:**
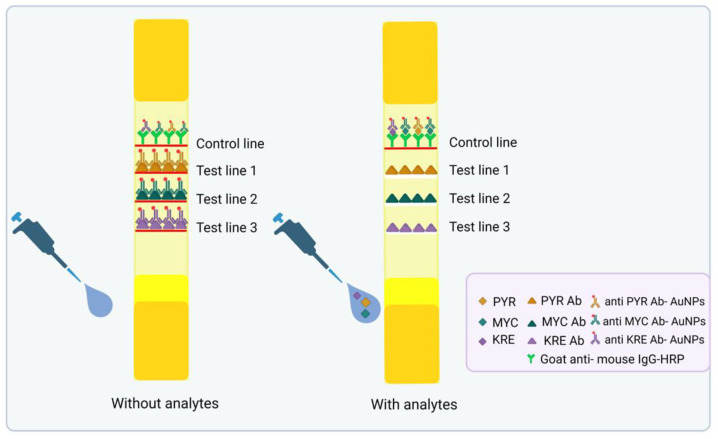
Schematic presentation of the method used for the simultaneous detection of fungicide in wheat [11]. Created in BioRender. LAMPROU, E. (2025) https://BioRender.com/t31y801 (accessed on 15 January 2025).

Antibody–gold nanoparticle conjugates (Ab–AuNPs) remain fundamental to LFA technology due to their simplicity and reliable results. Using simple colorimetry, Tsai et al. developed a multitarget assay for periprosthetic joint infection (PJI) detection in 2019, using alpha-defensin and C-reactive protein (CRP) as biomarkers, achieving 90% diagnostic accuracy. The method was overall simple, starting with premixing the AuNP-labeled antibodies, which were targeting the two biomarkers, followed by the collection of synovial fluid and the employment of a stacking pad design for enhanced detection sensitivity. The multitarget lateral flow immunoassay (LFIA) generated visually interpretable results as red-colored lines appeared on the test strip [7]. Chen et al. employed this method as well in 2016, for the simultaneous detection of mycotoxins (aflatoxin B1, zearalenone, and ochratoxin A) in grains, with limits well below regulatory thresholds. Specifically, the calculated limit of detection (LOD) values were 0.10–0.13 μg/kg for aflatoxin B1, 0.42–0.46 μg/kg for zearalenone, and 0.19–0.24 μg/kg for ochratoxin A [8]. Zhao and his team innovated further in 2022 by using distinct gold nanoparticles to differentiate pectin structural properties with an LOD of 0.02 μg/mL. In detail, they utilized four capture antibodies and two detection antibodies labeled distinctly with gold nanospheres and gold nano-urchins. Each detection antibody recognized different structural features of the polysaccharide, resulting in the appearance of different colors at the test zones. This multiplexing capability enabled the discrimination of various structural properties of pectin (LM19, LM20, JIM5, or JIM7), based on their degree and pattern of methyl-esterification [9]. Lee et al. enhanced, in 2015, the sensitivity for detecting AIDS, hepatitis A, and hepatitis C by integrating proteinticles as 3D probes, displaying viral antigens and protein-A conjugated AuNPs as reporters. The 3D probes, which displayed antigenic peptides with homogenous orientation and conformation, minimized issues like clustering, achieving 100% diagnostic sensitivity, with the use of calibrated densitometers and image analysis software [10]. Lin et al. developed in 2022 an LFIA for fungicide detection in wheat. They prepared three highly sensitive monoclonal antibodies (mAbs) by immunizing BALB/c mice with protein conjugates. Using the mAbs and AuNPs as reporters, very low limits of detection were achieved. The method is shown in Figure 2 [11]. Li et al. developed a simple LFA in 2024 for detecting SARS-CoV-2 variants and influenza A viruses, achieving detection limits of 4.88 ng/mL and 2.44 ng/mL, respectively, and showing 100% compliance with commercial kits [12]. Yang and his team used a similar strategy back in 2015 for diagnosing foot-and-mouth disease serotypes in swab samples [13], while Xu et al. designed a multiplex immunochromatographic strip in 2023 for detecting six steroid hormones in chicken and pork using the same principle and reaching limits of quantification between 0.32 and 0.64 μg/kg [14]. Yonekita’s team, in 2013, detected three serogroups of Shiga toxin-producing *E. coli* at low concentrations in food. They presented an LFIA that combined antimicrobial peptides (AMPs) labeled with colloidal AuNPs and a target-specific Ab immobilized on a nitrocellulose membrane. With this LFIA, *E. coli* O157, O26, and O111 were detected at very low levels (6.3, 2.9, and 5.6 colony-forming units—CFUs—per 25 g of ground beef, respectively) [15]. Shin et al. in 2024 utilized a multiplex colorimetric lateral flow immunoassay (CLFIA) based on the principles of antigen–antibody interactions and the unique optical properties of plasmon-controlled metal–silica isoform nanocomposites (PINs). In this assay, specific antibodies conjugated to PINs were bound to their corresponding analytes—intercellular adhesion molecule 1 (ICAM1), carbohydrate antigen 19-9 (CA19-9), and prostate-specific antigen (PSA), which are indicative of gastric, pancreatic, and prostate cancers, respectively. The bound complexes were then captured by immobilized antibodies on the test lines, resulting in a color formation that could be visually detected, with limits of detection of 6.65 ng/mL for ICAM1, 0.04 U/mL for CA19-9, and 0.12 ng/mL for PSA. This test significantly enhanced diagnostic sensitivity [16].

Competitive inhibition assays were effectively employed in food safety applications. A dual LFA utilized red gold nanospheres and blue gold nanoflowers (AuFl) for the simultaneous visual identification of bisphenol A (BPA) and dimethyl phthalate (DMP). The principle of the assay was based on a competitive reaction between contaminants in the sample and immobilized conjugates on the corresponding antigens on a nitrocellulose membrane. Monoclonal antibodies (mAb) conjugated with AuNPs and AuFl served as detection reagents. So, in the presence of the targets, the AuNP–mAbDMP and AuFl–mAbBPA conjugates bound to the analytes in solution, reducing the availability of the conjugates to interact with the immobilized capture reagents on the test strip and leading to decreased color intensity of the test lines. Following this principle, the assay achieved detection limits of 0.67 ng/mL and 2.22 ng/mL, for BPA and DMP, respectively [17]. Another LFIA focused on competitive inhibition reaction and the mycotoxins deoxynivalenol, fumonisin B1, and aflatoxin B1, which were detected at levels as low as 10 ng/mL in grain samples, using only AuNPs as reporters [18].

Signal amplification methods have been essential in improving the detectability and detection limits of LFAs. A good example is the one-step copper deposition-induced signal amplification method (osa-LFIA) using AuNPs as catalysts. In detail, the osa-LFIA featured a 3D flow channel design that was created using wax printing to form hydrophobic areas on cellulose paper, incorporating pre-dried copper sulfate and ascorbic acid. So, when the test sample flowed through the device, the AuNPs catalyzed the reduction of Cu^2+^ to Cu^+^ (CuO), which was then deposited onto the test line, amplifying the signal and darkening the color. This technique improved the detection sensitivity for *Staphylococcus aureus* (protein A) and *Pseudomonas aeruginosa* (exotoxin A), reducing detection limits from 0.3 μg/mL to 0.03 μg/mL for protein A and from 0.1 μg/mL to 0.01 μg/mL for exotoxin A in synovial fluid [19]. In an alternative approach, Yin et al. in 2024 enhanced antibody orientation using zinc-based nanocomposites in cascaded antibody directionality (CAbD) strategy to detect nitrofurazone and furazolidone metabolites in fish and honey samples. This technique resulted in ultrasensitive detection, achieving LODs of 0.106 ng/mL for nitrofurazone and 0.060 ng/mL for furazolidone [20].

Another example of exploiting colorimetric properties of nanoparticles is the use of different, than AuNPs, colored reporters. In that context, a multi-chromatic multi-component LFIA was developed, which utilized various colored microspheres as signal probes to detect specific analytes through antigen–antibody interactions. The assay was designed to detect multiple target proteins, indicated by the appearance of distinct colored bands corresponding to CP4, Bt, and PAT proteins. The optimization of assay conditions, including the concentration of immobilized antibodies, enhanced detectability, achieving LODs of 7.8 ng/mL for CP4 and 2.5 ng/mL for the other proteins in genetically modified crops within 10 min [21]. In another approach, a multiplex LFIA was reported for the detection of the allergens casein (CAS), ovalbumin (OVA), and hazelnut proteins (HNP) in food samples. The method involved the formation of a sandwich complex, where the allergens were captured by coated antibodies on the membrane, followed by detection antibodies labeled with colored nanoparticles (AgNPs, spherical AuNPs, and desert rose-like AuNPs), each producing distinct color lines (yellow, magenta, and cyan, respectively) for easy identification. The visual limit of detection for OVA and CAS was estimated at 1 mg/L, while for hazelnut extract it was 1% [22].

### 2.2. SERS-Based Lateral Flow Immunoassays

SERS-based LFAs represent a significant advancement over traditional LFAs, offering ultrasensitive and quantitative detection of biomolecules. A wide range of materials is used to create nanotags that enhance the SERS signal, often combined with magnetic particles to facilitate analyte detection in complex matrices. Below, recent examples of SERS-based LFΙAs and their potential applications are presented.

Chen and his team introduced an SERS-based LFΙA for the simultaneous detection of six mycotoxins on a single strip [23]. The novelty of this work lay in exploiting the multiplex labeling capability of SERS assays. In fact, two distinct Raman reporter molecules were used to label the synthesized Au@Ag core-shell nanoparticles, creating SERS detection nanoprobes. Six hapten–protein conjugates were deposited across the three test lines on the nitrocellulose membrane, with two conjugates on each line serving as capture probes. The target antigens were subsequently detected by the SERS nanoprobe conjugates. The optimized SERS-based LFΙA achieved detection limits ranging from 0.96 pg/mL to 0.26 ng/mL within just 20 min. The assay was validated using spiked samples and compared with conventional methods, demonstrating good agreement and reliability.

In an alternative SERS-based approach, silver-core and gold-shell bimetallic nanoparticles were utilized as SERS nanotags for the quantitative detection of cardiac biomarkers, enabling the early diagnosis of acute myocardial infarction [24]. This method followed the classical sandwich-type immunoassay format, where the biomarkers were first captured by immobilized specific antibodies and subsequently detected using the SERS nanotags. By leveraging the wide linear dynamic range of the assay, the method required no sample pretreatment and achieved detection limits at the picogram-per-mL level. The approach was compared with the laboratory standard chemiluminescence immunoassay and demonstrated strong agreement with it. Furthermore, the scalability of the assay for more analytes suggests significant potential for developing robust multiplex SERS-based LFAs in the future.

An SERS-based immunoassay was developed for the detection of infection biomarkers. This innovative SERS-based LFA utilized gold-functionalized magnetic nanoparticles as nanotags [25]. These nanotags were conjugated with specific antibodies and added to the sample, allowing all target biomarkers to be captured. This approach enabled target enrichment and signal improvement. The captured targets were then immobilized on the test lines of the LFA and detected using the SERS assay principle. The assay demonstrated high sensitivity, achieving detection limits of 0.1 ng/mL for serum amyloid A and 0.01 ng/mL for C-reactive protein. Furthermore, the SERS-based immunoassay was successfully applied to real whole blood samples without the need for additional pretreatment, showcasing the sensor’s efficacy in complex biological matrices.

In a similar approach, magnetic enrichment was employed for the detection of four veterinary drugs using an SERS-based lateral flow immunoassay in untreated complex samples [26]. In this study, multilayered magnetic-core dual-shell nanoparticles were assembled, consisting of superparamagnetic Fe_3_O_4_ cores coated with two layers of Au@Ag satellites. These nanoparticles were conjugated with specific antibodies for the target drugs and added directly to unprocessed samples to enrich the analytes and facilitate an efficient separation step. A competitive principle was used on the LFA strip, where the analytes that had been pre-captured by the SERS nanotags replaced the competitive antigens of the test lines of the strip. The multiplexing capability of the SERS assay enabled the simultaneous detection of two target molecules on each of the two test lines. The assay achieved a detection limit in the pg/mL range within 35 min, which was 10 to 400 times more sensitive than traditional AuNP-based LFAs.

Sheng et al. utilized alternative materials and a sophisticated architecture to develop SERS nanotags for an SERS-based LFA capable of simultaneously detecting three pesticide residues [27]. More specifically, silver-core and gold-shell nanotags were prepared, with 4-nitrothiophenol (4-NTP) encapsulated between the two metal layers to enhance the SERS signal. These nanotags were conjugated with specific antibodies for each of the three pesticides. The targets were captured on the nitrocellulose membrane of the strip following the standard LFA sandwich-type immunoassay principle. For their detection, streptavidin (SA)-labeled single-stranded DNA (ssDNA) was used to bind both the specific Ab-conjugated nanotags and the bare nanotags, leading to further signal improvement. This design enabled the assay to achieve a detection limit as low as 0.00015 ng/mL for chlorothalonil and 0.001 ng/mL for imidacloprid, while no data were provided for the third pesticide. The sensor was tested with environmental and food samples, showing excellent correlation with conventional gas chromatography methods for detecting pesticide residues. This demonstrated the potential of the multianalyte sensor for practical applications in environmental monitoring and food safety.

Another LFIA utilizing a dual detection system, SERS and photothermal signals, was developed for the simultaneous detection of influenza A, influenza B, and SARS-CoV-2 viruses [28]. The assay employed gold-core–silver-shell bimetallic nanoparticle conjugated with specific antibodies, each targeting one of the diseases. The targets were captured on three test lines of the LFIA strip and were detected by the conjugates. By integrating dual detection modes (SERS signals and photothermal signals), the sensor achieved a detection limit down to the pg/mL level. This novel approach, concerning the dual model detection, was successfully applied to real human samples, demonstrating strong concordance with commercial diagnostic assays.

In addition, multicolor SERS nanotags have enabled the simultaneous detection of dual analytes on a single test line in LFAs. Gao and collaborators introduced a novel SERS-encoded lateral flow immunoassay for the simultaneous detection of two model targets: carbendazim and imidacloprid [29]. Two distinct nanoprobes were designed, each encoding both the color and SERS signal information within a single LFA test. The first nanoprobe utilized Prussian blue-coated AuNPs, while the second employed 4-mercaptobenzonitrile-coated AuNPs, producing blue and red color signals, respectively, along with distinctive Raman spectra. These nanoprobes were conjugated with specific antibodies to target the analytes, which were detected via the lateral flow immunoassay principle. Detection results were visible through the color changes and measurable via SERS signals, achieving a detection limit in the microgram-per-milliliter range. This dual-mode detection paves the way for future applications in the simultaneous identification of multiple biomolecules.

Exploiting the same LFIA principle as described above, another group developed and reported a novel detection system [30]. They used polystyrene microspheres coated with AuNPs and Raman probes serving as SERS tags. The microcavities of the SERS tags enhanced the assay signal through the localized surface plasmon resonance of the AuNPs. These sensitive tags were applied for the dual simultaneous detection of heart failure and infarct biomarkers, achieving a limit of detection in the pg/mL range within 15 min—up to 20 times lower than conventional AuNP-based LFAs.

Alzheimer’s disease (AD) biomarkers were also detected with an impressive limit of detection down to the fg/mL range [31]. In this approach, the simultaneous detection of the biomarkers followed the immunoassay principle. The innovation in this work was the SERS probes, which were designed using silica nanoparticles with AuNPs, conjugated with specific antibodies, which enhanced the SERS signal through their porous structure. Additionally, the test strip was fabricated using an alternative material, inverse opal nitrocellulose, which reduced analysis costs while increasing detectability. The limits of detection were 15.3 and 16.8 fg/mL for the two biomarkers.

### 2.3. Fluorescent Lateral Flow Immunoassays

Quantum dots (QDs) have become a cornerstone in lateral flow immunoassays due to their ability to emit fluorescence at different wavelengths, allowing for highly sensitive and multiplexed detection. Cadmium-free indium phosphide (InP) QDs were conjugated with monoclonal antibodies to detect mycotoxins zearalenone (ZEN) and deoxynivalenol (DON). The QDs were functionalized with SiO_2_ (silica) shells to enhance stability, then activated with EDC (1-Ethyl-3-(3-dimethylaminopropyl)carbodiimide) and sulfo-NHS (sulfo-N-hydroxysuccinimide), to conjugate with antibodies against ZEN and DON. In the presence of the target analyte, the fluorescence intensity in the test zone decreased due to competition between the analyte and analyte–protein conjugates for antibody bonding, allowing for quantification. The method achieved cutoff values of 50 μg/kg for ZEN and 500 μg/kg for DON in maize and wheat samples [32]. Similarly, Cao et al. in 2022 used dual-color QDs in a biosensor to detect gastric cancer biomarkers (PG I and PG II). The conjugated QDs emitted at different wavelengths, and their fluorescence intensity ratios were then analyzed to quantify the concentrations of the targets in serum. This dual-color approach allowed for simultaneous, multiplexed detection on a single test line, enhancing the accuracy of the assay while reducing background noise. The detection limits for PG I and PG II were 0.29 ng/mL and 0.66 ng/mL, respectively [33]. Xiong and his team further in the same year developed a method that employed SiO_2_@QD nanocomposites for the simultaneous detection of ractopamine and salbutamol in swine urine and pork. These composites provided excellent fluorescence stability and sensitive detection with an LOD of 0.007 ng/mL for ractopamine and 0.032 ng/mL for salbutamol. The QDs were bound to the analytes in a competitive immunoassay, while the fluorescence intensity was related to the concentration of the analytes [34]. Huang et al. developed in 2024 a multiplex QD-based assay for detecting food allergens like ovalbumin and tropomyosin in a single assay, achieving detection limits of 0.05 μg/mL and 0.5 μg/mL, respectively [35]. An LFA that utilized dendritic mesoporous silica nanoparticles functionalized with carbon dots was designed to detect specific analytes through a straightforward and rapid process. The method involved applying a sample containing the target analytes, which were procalcitonin (PCT), carbohydrate antigen 19-9 (CA19-9), and alpha-fetoprotein (AFP), onto a sample pad treated with antibodies specific to the analytes. Upon interaction, the bound analytes were captured on a test line, generating a fluorescent signal that was further quantified with ImageJ software (ImageJ 1.53t). The assay demonstrated remarkable limits of detection of approximately 0.01 ng/mL for PCT, 0.1 U/mL for CA19-9, and 0.01 ng/mL for AFP, highlighting its high sensitivity and potential for effective clinical diagnostics [36].

**Figure 3:**
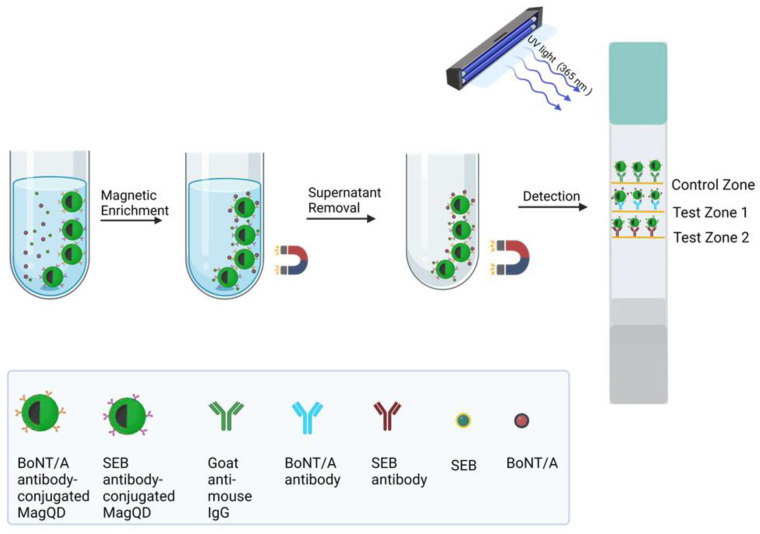
Visual summary of the LFA developed for the detection of toxins in food samples using magnetic QD NPs as reporters [38]. Created in BioRender. LAMPROU, E. (2025) https://BioRender.com/t31y801 (accessed on 15 January 2025).

Nanoclusters and nanocomposites have significantly enhanced the detectability and stability of LFAs. A multiplex LFIA (AuNCs–MLFIA) was designed, using green-emitting gold nanoclusters for the detection of clenbuterol (Clen) and ractopamine (RAC) in swine urine. The gold nanoclusters provided strong fluorescence in the green spectrum, enabling sensitive detection of both analytes, with LODs of 0.003 mg/L for clenbuterol and 0.023 mg/L for ractopamine [37]. Wang et al. introduced in 2019 magnetic quantum dot nanoparticles (MagQD NPs) for detecting protein toxins (BoNT/A and SEB) in food samples. The magnetic properties of the nanoparticles facilitated the separation of the toxin–antibody complexes with the use of an external magnet and ensured high specificity and quantification. When bound to the targets, the QDs emitted fluorescence, allowing simultaneous detection, as shown in Figure 3. The system achieved detection limits of 2.52 pg/mL for BoNT/A and 2.86 pg/mL for SEB, in complex samples like milk and grape juice [38].

Moving forward, a biotin-enriched dendritic mesoporous silica nanoparticle-based LFA (BDMSNs-MLFIA) was introduced, for detecting ovarian cancer biomarkers (CA125 and HE4) in human serum. The high affinity of the biotin–streptavidin system, combined with aggregation-induced emission molecules, significantly improved the sensitivity and accuracy of detection. The BDMSNs-MLFIA achieved detection limits of 5 U/mL for CA125 and 5 pM for HE4. Moreover, this approach provided a correlation coefficient of over 98% after comparison with commercial chemiluminescence methods, proving its strong potential [39]. A multiplex fluorescence bead-based LFIA (mFB-LFIA) was also developed for the simultaneous detection of three swine coronaviruses, PDCoV, TGEV, and PEDV, in fecal samples. The method used europium nanoparticles (EuNPs) as reporters and sandwich-type immunoassay for qualitative and quantitative detection. Pretreatment of the sample and conjugate pads, along with a filter pad, reduced interference from fecal matter, enhancing detectability. Immune complexes were captured on three separate test lines, corresponding to the viruses, with the fluorescent signal measured to indicate viral presence. This method demonstrated high detectability, stability, and reproducibility, making it a valuable tool for swine coronavirus diagnostics [40].

**Figure 4:**
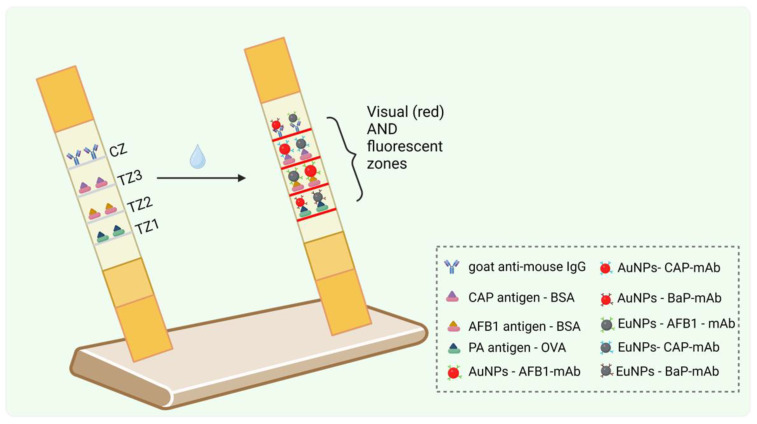
Assay principle of the novel dual-readout LFA for the detection of pollutants. CZ, control zone; TZ, test zone [44]. Created in BioRender. LAMPROU, E. (2025) https://BioRender.com/t31y801 (accessed on 15 January 2025).

Dual-mode assays combine fluorescent and colorimetric detection to improve detectability and versatility. In this direction, a dual-mode LFA was developed, combining MoS_2_ nanosheets with QDs for detecting agricultural drug residues, such as clothanidin, carbendazim, kanamycin, and chloramphenicol. The dual-color labels allowed for fluorescence-based LODs of 1.53–4.88 pg/mL and colorimetric LODs of 0.12–0.36 ng/mL, enabling rapid screening and quantitative detection within 15 min [41]. Zhang et al. in 2024 presented a multicolor LFIA using aggregation-induced emission nanoparticles (AIENPs) for the detection of aflatoxin B1 (AFB1) and zearalenone (ZEN) in corn and wheat samples. The AIENPs emitted distinct colors, each corresponding to a specific analyte, allowing for clear differentiation of the test lines. This method achieved detection limits of 6.12 pg/mL for AFB1 and 26 pg/mL for ZEN [42]. A bimodal-type multiplexed LFIA was created for detecting multiple analytes, specifically ractopamine (RAC) and clenbuterol (CLE). The method employed a spatial separation traffic light-typed colorimetric-fluorescence dual-response assay (STCFD) that integrated two distinct fluorescent donors—AuNCs–BSA (red fluorescence) and Arg/ATT–AuNCs (green fluorescence)—with a colorimetric signal from the amphiphilic peptide nanoparticles (APNPs). The LFIA strip was designed with two test zones where specific antigen–antibody interactions occur, leading to the formation of visible signals under both UV light and the naked eye. The assays’ performance was enhanced by optimizing the spectral overlap between the fluorescence donors and the colorimetric signals, allowing for simultaneous detection, with the results visually interpreted through distinct color changes (red, green, and yellow) and fluorescence intensity variation, resulting in multitarget monitoring. The limits of detection for this assay were 0.013 ng/mL for RAC and 0.152 ng/mL for CLE, demonstrating its capability for ultrasensitive analysis [43]. Next, a time-resolved fluorescence/visual dual-readout lateral flow immunoassay (tdLFIA) was presented, which employed a competitive immunoassay format where the target analytes (aflatoxin B1, benzo(α)pyrene, and capsaicin) competed with their respective conjugated antigens for binding to specific monoclonal antibodies immobilized on a test strip. In this quite simple assay, when a sample containing the analytes was applied, the analytes bound to the antibodies, displacing the conjugated antigens that were conjugated to AuNPs (for visual detection) and Eu^3+^-labeled fluorescent nanospheres (for fluorescent detection) (Figure 4). The resulting signal intensity, which increased with increasing concentration of the target analytes, was valuated both visually and through fluorescence, allowing for rapid and sensitive quantification of the contaminants in edible oils, with detection limits of 0.003 ng/mL for AFB1, 0.6 ng/mL for BaP, and 0.01 ng/mL for CAP [44].

A multiplex competitive LFIA (cLFIA) based on the inner filter effect (IFE) between flower-like gold nanoparticles (AuNFs) and red-emitting QDs was developed for the simultaneous detection of aflatoxin B1 (AFB1), ochratoxin A (OTA), and zearalenone (ZEN) in maize. AuNPs served as quenchers of the QDs. The antigens were sprayed at three test zones to develop the competitive immunoassay, along with BSA-coated QDs. AuNFs were coated with antibodies specific to the antigens and SA. AuNFs, in the absence of the antigens in the sample, were captured at the test lines, and the accumulation of the AuNFs resulted in QD fluorescence quenching. SA-QDs were captured also at the control zone by immobilized biotinylated BSA. In the presence of the mycotoxins in the sample, they competed for the attachment to the AuNFs, turning on fluorescence at the test lines. This system had LODs of 0.005 μg/L for AFB1, 0.04 μg/L for OTA, and 0.4 μg/L for ZEN. After spiking of the antigens in maize samples, the recoveries were calculated to be 72.0–92.4% for AFB1, 79.2–105.60% for OTA, and 82.0–105.00% for ZEN. All coefficients of variation (CVs) were below 15.5% [45].

Multiplexed LFAs combined with automation have enabled high-throughput and rapid diagnostics for various applications. For instance, Rong et al. in 2021 developed a multiplexed diagnostic platform that integrated a multichannel cartridge with a fluorescent lateral flow assay for HIV, Treponema pallidum, HCV, and HBsAg detection. The system employed quantum nanobeads conjugated with target-specific antibodies, with fluorescence signals detected and analyzed by a custom optical reader, providing high detectability (LODs of 0.11 novo cellulase unit (NCU)/mL for HIV Ab, 0.62 IU/L for TP Ab, 0.14 NCU/mL for HCV Ab, and 0.22 IU/mL for HBsAg within 20 min) and specificity in a low-cost format for POC use [46]. Chen et al. in 2022 adopted this approach for detecting respiratory viruses like SARS-CoV-2, influenza A virus (IAV), influenza B virus (IBV), and adenovirus (ADV), with detection limits of 0.01 ng/mL, 0.40 ng/mL, 0.31 ng/mL, and 30 pfu/mL, respectively [47].

### 2.4. Nanozymes

Recent advancements in nanozyme-based LFIAs have significantly improved detectability, detection range, and versatility for detecting hazardous substances in food safety. A dual-readout multiplex LFA utilizing magnetic Prussian blue nanozymes (MPBNs) enabled simultaneous detection of ractopamine (RAC) and clenbuterol (CLE). MPBN acted as a bifunctional signal tag, enhancing both visual and quantitative analysis through a competition-type immunoreaction. This approach achieved a two-fold increase in detection range, with recoveries ranging from 84.01% to 119.94% and calculated limits of detection at 0.20 ng/mL for CLE and 0.12 ng/mL for RAC [48]. Similarly, the development of a mussel-inspired Fe-based tannic acid nanozyme (FTAN) provided dual-readout capabilities, leveraging its inherent colorimetric (from the FTANs’ inherent color) and catalytic properties (from its enzyme-mimicking activity) to enhance the detection of RAC and CLE. This method, which offered high coupling efficiency with antibodies, was effective for on-site monitoring of food contaminants in an environmentally friendly and cost-effective manner, giving calculated LODs of 0.015 ng/mL for RAC and 0.156 ng/mL for CLE [49]. For veterinary drug residues, a Fe-Au@Pt magnetic nanozyme enabled ultrasensitive detection of gentamicin, streptomycin, and clenbuterol in food samples, achieving detection limits as low as 10.1 pg/mL for gentamicin, 6.3 pg/mL for streptomycin, and 1.1 pg/mL for clenbuterol and demonstrating recovery rates of 80.5% to 116.3% in real samples, such as honey, milk, and pork. In this system, the Ab-modified Fe-Au@Pt nanozyme bound to target drug residues in the sample, forming immunocomplexes, which reduced the binding of free nanozymes to the test zones and led to a weaker colorimetric signal. This competitive immunoassay technique amplified the detection limit by 30–300 times compared with conventional methods, highlighting its potential for food safety applications [50].

### 2.5. Smartphone-Based Lateral Flow Immunoassays

Smartphones play a crucial role in enhancing LFAs by serving as portable, user-friendly platforms for real-time data interpretation and analysis. With their widespread accessibility, smartphones simplify sensor readouts and support applications for advanced data processing. This enables qualitative analysis and enhances the sensitivity of LFAs. By leveraging advanced camera technology and AI algorithms, smartphones automate the reading of test results, improving accuracy. Moreover, their connectivity features facilitate data sharing and efficient monitoring of results by experts. Several representative examples from the recent literature are presented below.

A decentralized approach for on-field testing was reported for the detection of venom from two dangerous snake species in Brazil [51]. This dual LFA-integrated smartphone technology provided semi-quantitative results. Carbon nanoparticles (CNPs) were conjugated with specific antibodies, allowing the target venom to be captured by an immobilized Ab on a nitrocellulose membrane. Detection was achieved using the CNPs–Ab conjugates, based on the sandwich ELISA assay principle. The LFA was successfully applied to spiked bodily fluids, including serum, plasma, and urine. The detection limits for both analytes ranged from 10 to 500 ng/mL.

Another LFIA was integrated with a smartphone for the quantitative analysis of mycotoxins [52]. The immunoassay utilized gold and platinum nanozymes conjugated with specific antibodies. These nanozymes catalyzed the oxidation of a peroxidase substrate, producing a colored enzymatic product that was quantified using a smartphone camera and a dedicated application. The sensitive dual mycotoxin assay, targeting deoxynivalenol and zearalenone, was analytically characterized and achieved detection limits of 0.24 ng/mL and 0.04 ng/mL, respectively—up to 400 times lower than conventional lateral flow assays using AuNPs.

In another study, a dual-detection LFIA combining AuNPs and time-resolved fluorescence microspheres (TRFMs) was developed for the simultaneous detection of five mycotoxins in cereal samples [53]. Specific antibodies for each mycotoxin were conjugated with both AuNPs and TRFMs to generate visual and fluorescent signals, respectively. Images of the LFIA strips were captured using a smartphone housed in a 3D-printed dark box and analyzed with a dedicated universal application designed for this assay, providing dual quantitative results for all five mycotoxins. The quantitative detection limits achieved with the smartphone-based quantification were 2 to 20 times lower than those based on simple visual detection. The dual-detection LFIA was validated against conventional detection methods, demonstrating its reliability and highlighting its potential for on-field mycotoxin testing in food samples.

A centrifugated LFA approach was developed for the multiplex detection of tetracycline residues [54]. Dual-emission fluorescent probes, consisting of carbon dots modified with europium anions, were designed for the determination of four tetracyclines. In the presence of target molecules, the fluorescence of the probes was quenched due to the inner filter effect and localized fluorescence resonance energy transfer (L-FRET) between the probes and the tetracyclines. Without additional modifications, the fluorescent probes were able to differentiate each target based on the distinct fluorescence changes corresponding to their quenching efficiency. The LFAs were integrated with a centrifugation system to control liquid flow through the strips and enhance assay sensitivity. Fluorescent strip images were captured using a smartphone, and RGB model variations were analyzed to interpret and characterize the device. The method achieved detection limits in the nanomolar range, and the integrated fluorescent sensor with centrifugation-enhanced LFAs was successfully applied to spiked water samples, yielding high recovery rates.

A simultaneous multiplex aptamer-based LFIA was developed for the detection of three distinct targets: mercury ions, ochratoxins, and *Salmonella* [55]. Specific aptamers were designed for each target and immobilized on the nitrocellulose membrane of the lateral flow strip. Multicolor upconversion nanoparticles (UCNPs) were conjugated with complementary single-stranded DNA to bind the aptamers. Upon target recognition, the presence of the target molecules led to a decrease in the fluorescent signal from the nanoparticles, creating a signal-OFF detection platform. The fluorescent images of the assay were captured using a smartphone-based portable device, and the results were analyzed using densitometric analysis in the RGB model. The detection achieved limits were 5 ppb for mercury ions, 3 ng/mL for ochratoxin, and 85 CFU/mL for *Salmonella*. The aptamer-based LFA was successfully applied to real water samples, demonstrating its utility for on-field testing.

Deep-learning-assisted detection is at the forefront of advancements in POC diagnostic devices. In a recent study, a zwitterionic magnetic immunochromatographic assay was developed for the multiplex diagnosis of proteins associated with SARS-CoV-2 and influenza A/B [56]. Magnetic nanoparticles (MNPs) were utilized as they offer higher versatility compared with conventional AuNPs and fluorescent probes, particularly due to their superior signal-to-noise ratio. The MNPs were functionalized with carboxyl groups and zwitterionic ligands, which served as specific probes for each target protein. The LFIA achieved high sensitivity and detection limits of 0.0062 ng/mL for SARS-CoV-2 and 0.0051 ng/mL and 0.0147 ng/mL for the N proteins of influenza A and influenza B, respectively, all within 12 min. Deep learning was integrated into this test, employing a trained algorithm to classify samples with high accuracy based on concentration and test positivity. This integration not only enhanced the analytical characterization of the assay but also facilitated its use in decentralized diagnostic settings.

Wang et al. developed a novel paper-based assay that leveraged the versatility and customizability of paper, combined with AuNPs conjugates [57]. Specifically, a vertical flow assay was designed for the simultaneous detection of three acute kidney injury (AKI) biomarkers within a single test. Capture antibodies were immobilized on the nitrocellulose membrane, connected with absorbent paper, to bind target molecules, which were subsequently detected using specific Ab–AuNPs conjugates. Results appeared within 15 min as red-colored spots in the presence of the target biomarkers, with the intensity of the color correlating to the target concentration. For quantitative analysis, a custom smartphone-based readout system was used to interpret the visual results, achieving a detection limit of approximately 1 ng/mL. The clinical applicability of this vertical paper-based immunoassay was demonstrated by successfully testing the three AKI biomarkers in clinical samples, highlighting its potential for rapid and reliable diagnostic applications.

Additive manufacturing, combined with the development of user-friendly applications and robust fluorescent nanocomposites, was integrated into the classic principles of LFAs. A 3D-printed case was designed to house optical and electrical components, enabling the miniaturization of a fluorescent reader [58]. This dual-platform system was developed for the detection of illegal drugs. Silica core quantum dot-shell nanocomposites, conjugated to specific antibodies, were employed as detection probes. Following the conventional lateral flow assay format, a capture Ab was used for target recognition, while the nanocomposites facilitated fluorescent detection. The resulting fluorescence signal was captured by a camera, and the recorded images were analyzed using a custom-built Android-compatible smartphone application. This dedicated application provided quantitative analysis of methamphetamine and tramadol, achieving detection limits of 0.11 ng/mL and 0.017 ng/mL, respectively.

## 3. Nucleic Acid-Based Lateral Flow Assays

DNA-based methods are widely used for species identification, facilitating authentication of several products, as well as applications in disease diagnosis, forensic science, environmental monitoring, and agriculture and food safety. Given its specificity and reliability, DNA sequences have become preferred analytes of interest for various methods. Target amplification, however, is required and represents a crucial step, along with sample pretreatment for DNA isolation, that both influences the detectability and prolongs the detection times of the methods. For multiplex DNA-based LFAs, multiplex PCR is usually the preferred approach for rapid and low-cost analysis. After the PCR amplification step, a multiplex LFA is a more useful alternative than other post-PCR detection methods, such as agarose gel electrophoresis or DNA sequencing, for time and cost saving, increasing also the simplicity of the analysis. LFAs, compared with agarose gel electrophoresis, provide simultaneous identification of the target DNA sequence [59]. In general, most amplified products are dually labeled during the amplification step, with two different haptens/proteins or other small molecules such as biotin, to enable detection on a lateral flow strip.

### 3.1. Polymerase Chain Reaction (PCR)-Based Amplification

**Figure 5:**
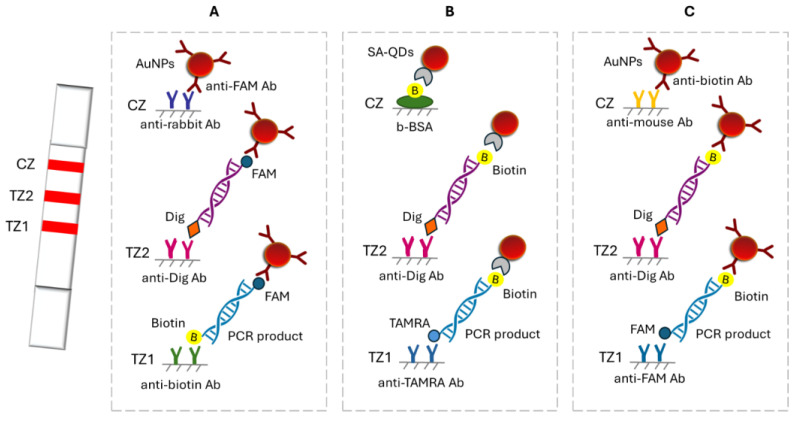
Configurations of double LFAs. PCR products are labeled either with fluorescein amidite (FAM) at one end and digoxigenin (Dig) or biotin (B) at the other end and detected by anti-FAM antibody–gold nanoparticles (Ab–AuNPs) (**A**) [59] or with biotin and Dig and or TAMRA (**B**) [1] or FAM (**C**) [61] and detected by streptavidin–quantum dots (SA–QDs) or antibiotin–AuNPs. TZ, test zone; CZ, control zone; Ab, antibody.

DNA barcoding is a widely accepted method for species identification that usually exploits the conserved regions of mitochondrial multicopy genes. DNA barcoding is combined with nucleic acid amplification techniques. In this context, a double LFA was developed for the simultaneous identification of two closely related shrimp species based on the Cytb gene, allowing shrimp adulteration detection, as fish adulteration is a major problem to the fisheries sector. The method involved a double PCR using species-specific primers labeled at one end with fluorescein (FAM) and at the other end with biotin or digoxigenin (Dig) to allow the discrimination of the two species with the LFA. After amplification, the PCR products were applied to the strip and were captured at two test zones by immobilized anti-biotin and anti-dig antibodies. The captured products were then detected by anti-FAM antibodies conjugated to AuNPs, forming two red lines. The excess of AuNPs was captured by immobilized antibodies, forming a third red line (Figure 5A). The double test showed very good specificity, while as low as 0.02% (0.01 ng) of adulteration was detected. The test was finally applied successfully to cooked and commercial samples [59]. A similar dual LFA was developed for the simultaneous detection of the bacteria *Escherichia coli* O157:H7 and *Salmonella Typhimurium*. Rapid and sensitive detection of various infections is very important. Similarly, a double PCR was deployed using specific primers labeled with biotin at one end and at the other end with Dig for *E. coli* and FAM for *S. Typhimurium*. The PCR products were captured at the test zones by immobilized anti-Dig and anti-FAM antibodies, while the detection was accomplished by streptavidin–AuNPs (SA–AuNPs). The LFAs provided LODs of 10^2^ CFU/25 g and 10 CFU/25 g of *E. coli* O157:H7 and *S. Typhimurium* in 100 min, respectively. Bacteria specimens were first treated with propidium monoazide (PMA) to identify dead bacteria and thus minimize false-positive results because PAM dye intercalates into the DNA strands only in injured and dead cells, where DNA is available. The samples were then filtered, and DNA was isolated only from the viable bacteria cells. During this procedure, the samples were also enriched, leading to increased detectability up to 10 times [60]. The simultaneous detection of SARS-CoV-2 and influenza B viruses was accomplished similarly with a dual strip and a dual PCR using primers labeled with biotin and Dig or TAMRA dye. The detection here was based on fluorescent QDs conjugated to SA (Figure 5B). The detection limits were 8.4 copies/mL for SARS-CoV-2 and 14.2 copies/mL for influenza B virus. The test showed very good reproducibility with CVs of 10.1% for SARS-CoV-2 and 4.9% for the influenza B virus and was developed as an effort to improve the detectability and specificity of existing LFIAs to detect these viruses [1]. Recently, more research groups combined AuNPs with PCR amplification to further enhance the specificity. Namely, Phopin et al. developed a duplex PCR–LFA with a similar configuration for detecting *Salmonella* and *Vibrio cholerae* in food samples. After the amplification step, the PCR products were premixed with the running buffer and added to the sample pad of the strip, where anti-biotin-coated AuNPs were pre-deposited. Visible red lines were formed at the test zones of the strip after 15–30 min, indicating the presence of the specific bacteria in the sample (Figure 5C). This protocol enhanced the usability and flexibility of the LFA and provided LODs of 10^6^ and 10^4^ CFU/mL in spiked raw oyster and chicken meat, respectively [61]. The second group, Moon et al., used an extended approach, with a multiplex PCR/LFA for the simultaneous identification of the bacteria *P. carotovorum brasilience* and *E. coli* O157:H7. This system worked by amplifying bacteria genes using primers labeled with FAM or Dig. The resulting amplicons were then mixed with an LFA dilution solution and applied to a dual LFA strip, where they were bound to immobilized species-specific Abs and detected by anti-FAM or anti-Dig AuNP conjugates. The limit of multiplex PCR using a portable PCR device and the subsequent LFA was found to be 1.26 CFU/mL for *P. carotovorum* subsp. *brasilience* and 13.7 CFU/mL for *E. coli* O157:H7 [62].

The optimization of the immobilization of nucleic acid sequences and oligonucleotides on the diagnostic membrane of LFAs was recently investigated to increase the simplicity of LFA construction and enhance the produced visual signal. The immobilization of DNA probes in the presence of high salt (0.8 M KCl) was tested and compared with the immobilization without the addition of extra salt. Immobilization was then investigated (i) at room temperature for 30 min, (ii) under UV light for 5 min with a total energy at 90 mJ/cm^2^, and (iii) at 60 °C for 10 min. The proposed LFA was applied for the simultaneous detection of three synthetic DNA targets corresponding to three different genes of the SARS-CoV-2 virus after asymmetric PCR. AuNPs coupled to anti-biotin Ab were used as reporters herein. The results showed an increase in the signal 14 times in the presence of salt and for 30-min incubation at room temperature compared with immobilization without salt. The LFAs showed good reproducibility, high specificity, and long-term storage stability, while an LOD of 300 aM was achieved [2].

**Figure 6:**
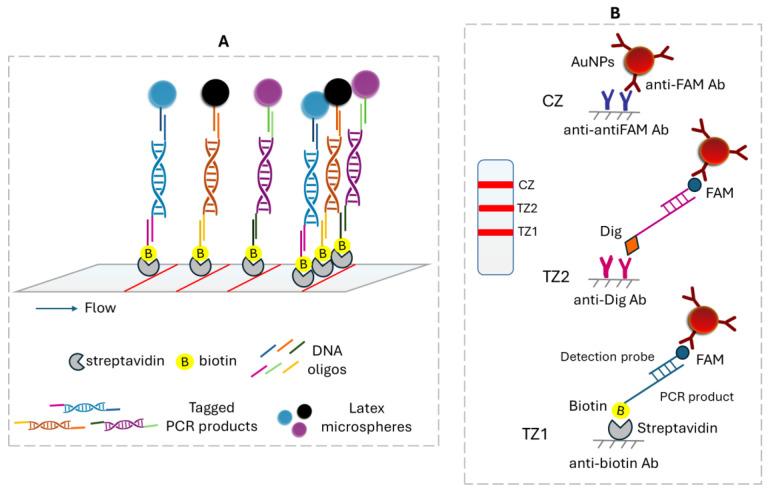
Principles of LFAs. (**A**) PCR products contained two tags at both ends. After application onto the strip, PCR products were captured by immobilized DNA recognition probes and detected through colored latex microspheres of different colors that were coupled to each target’s specific DNA probe [63]. (**B**) PCRs were labeled with biotin (B) or digoxigenin (Dig) and hybridized to complementary probes prior to application onto the strip with specific DNA probes that carried a fluorescein amidite (FAM) moiety at one end. The hybrids were detected through capturing by immobilized anti-biotin and anti-Dig antibodies (Ab) and by anti-FAM Ab–AuNPs. AuNPs, gold nanoparticles; TZ, control zone; CZ, control zone [79].

A triple-color LFA combined with multiplex PCR was developed for the identification of beef, pork, and chicken meat. Meat species identification is crucial as meat adulteration is a major problem in the food industry. Therefore, analytical techniques and biosensors for multiplex testing are in high demand for food quality assessment. In this work, colored latex microspheres were used as reporters to enhance spatial discrimination on the strip, compared with AuNPs. Microspheres of different colors were coupled to recognition DNA probes to allow the discrimination of specific DNA sequences. A multiplex PCR was performed using specific primers of the three animal species tested. The PCR products were flanked with oligonucleotide sequences at both ends. One end was complementary to the recognition probe coupled to the microspheres specific to each species, and the other end was complementary to the capture probe immobilized through streptavidin at the test zone of the LFA. Three biotinylated capture probes were used to construct three test lines through streptavidin, corresponding to the three meat species. The color formed at the test line denoted the presence of the specific animal species in the sample (Figure 6A). This novel LFA gave an LOD of 0.1% of adulteration with high specificity between other species. The reproducibility data indicated CVs in the range of 5–13% across all species tested, reflecting a consistent and reliable performance [63]. Moreover, a dual strip was developed for the quantitative detection of SARS-CoV-2 incorporating a DNA internal standard for improved accuracy. The internal standard had the same DNA sequence of amplified product as that of the nucleoprotein gene but differed in a 24-bp segment located in the center of the sequence. This segment was exploited for the detection of PCR products on the strip using complementary detection DNA probes. Nasopharyngeal samples were subjected to RT-PCR in the presence of a known amount of the DNA internal standard. The SARS-CoV-2 DNA sequence and the internal standard were amplified using the same PCR primers and hybridized to detection probes that carried a specific tag oligonucleotide tail. The hybrids were then visualized by immobilized anti-tag sequences and anti-biotin Ab-AuNPs conjugates as red spots. Finally, the color intensity of each spot was measured by the ImageJ software, and a calibration curve was constructed. This dual strip had an LOD of one copy of plasmid DNA corresponding to the SARS-CoV-2 DNA sequence and CVs < 0.7% [64].

A multiplex LFA was also developed for the detection of miRNAs related to colorectal cancer. This system combined stem-loop RT and asymmetric PCR using forward primers with extended sequences to increase the size of the amplified miRNA sequence. The asymmetric PCR products were biotinylated through a universal reverse primer and carried a DNA barcode through the specific forward primers. Multiplex analysis of three miRNAs was accomplished using different DNA barcodes. Herein, the harness of DNA barcodes instead of haptens as labels of the primers during PCR enhanced the versatility and multiplexing potential of LFAs. The PCR products were captured onto the lateral flow strip, through the DNA barcodes, to immobilize complementary DNA sequences and visualized by SA–AuNPs. The proposed system successfully detected miR-92a, miR-141, and miR-345 that were overexpressed in colorectal cancer exosomes. The proposed LFA, after clinical sample analysis, showed a sensitivity and specificity of 95.24% and 100.0%, respectively [65].

Finally, PCR was combined with multi-allelic discrimination reactions (MADRs) and multiplex LFAs for the simultaneous detection of single nucleotide polymorphisms (SNPs) in various targets for clinical diagnostics or food authenticity testing. For this purpose, a quadruple LFA was developed for the simultaneous detection of three major mutations of the KRAS gene in circulating tumor DNA (ctDNA), related to colorectal cancer, along with the normal gene. The method included isolation of cell-free DNA from plasma samples, amplification of the KRAS gene by PCR, multiplex allele discrimination reaction (PEXT), and detection of the extended products with a multiplex LFA. During the PEXT reaction, a mixture of PEXT primers that corresponded to each allele were used. These primers had the same sequence but contained a different base at the 3′ end corresponding to the mutation and a different tag tail at the 5′ end. After MADR, the extended products were visualized as spots by immobilization complementary to the tag sequences and SA–AuNPs. The multiplex strip showed high specificity and reproducibility (CVs 0.5–2.8%) [66]. Similar approaches with the use of antibiotin–AuNPs were developed for the simultaneous detection of 2 common SNPs (4 alleles) of the Toll-like receptor 4 (TLR4) gene related to the immune system [67] and the MBL2 gene [68], 10 SNPs in the HBA1 and HBA2 genes related to a-thalassemia using two strips that could detect 5 SNPs simultaneously and thus 10 alleles [69], and the Wilson disease-related ATP7B gene [70]. Also, the three and four most frequent SNPs were detected in the gene of coagulation factor V, known as FV Leiden, in the FII (prothrombin, PTH) gene and in the methylenetetrahydrofolate reductase (MTHFR) gene using two separate strips [71]; four SNPs in each of two strips in the SIRT1 gene related to sporadic Parkinson disease [72]; and five SNPs in a single multiplex strip in MBL2, TLR4, and JAK2 genes [73]. Last, similar strategies were exploited for the development of a multi-species DNA strip sensor for the simultaneous recognition of seven plant species in edible oils for olive oil adulteration assessment [74] and multi-allelic sensors for the detection of four SNPs (two SNPs—four alleles per strip) in order to identify the olive variety for olive oil authenticity testing [75]. All the lateral tests showed very good specificity and excellent reproducibility.

### 3.2. Loop-Mediated Isothermal Amplification (LAMP)

Isothermal amplification techniques have attracted the research community’s interest because they provide amplification of nucleic acid sequences at constant and low temperature in reduced time without the requirement of a thermocycler. They are based on multiple strand displacement reactions. Also, in combination with portable devices, such as LFAs, they are ideal tools for point-of-care testing and in field detection. However, multiplex LAMP products are difficult to detect during the reaction when more than one amplicon is produced. Therefore, simple methods for further analysis of LAMP products are needed. Several methods have been introduced with an attempt to increase the number of analytes that are simultaneously amplified and detected within a single strip. On the other hand, LAMP provides high specificity because it uses four or six specific primers for amplification.

A multiplex reverse transcription loop-mediated isothermal amplification (RT-LAMP) was combined with a nanoparticle-based lateral flow biosensor for the diagnosis of COVID-19. Using two sets of LAMP primers, two genes of the SARS-CoV-2 virus (ORF1ab and N nucleoprotein) were simultaneously amplified. By detecting two targets, the reliability of the test result was increased. The LAMP products here were also labeled with FITC or Dig, and biotin and detected with the strip by immobilized anti-FITC and anti-Dig antibodies, while the products were visualized by polymer nanoparticles conjugated to SA. Specificity was finally assessed by using synthetic nucleic acid sequences, viruses, bacteria, and fungi. The LOD of this strip was 12 copies for each target, while no cross-reactivity was observed. The analytical sensitivity and specificity were both 100%, and the total analysis time was 1 h [76]. A similar approach was developed for the simultaneous detection of *Salmonella* spp. and *Cronobacter* spp. with a dual LAMP reaction. Foodborne pathogens constitute a public health issue, and their rapid and multiplex detection is of great importance. In this method, the LAMP amplicons were labeled with Dig or biotin, and FITC and detection was accomplished by AuNPs conjugated to anti-FITC antibodies. The test zones were constructed by immobilization of anti-Dig Ab and SA. This configuration showed LODs of 4.3 CFU/g and 2.8 CFU/g for *Salmonella* spp. and *Cronobacter* spp., respectively, in powdered infant formula. The test showed high specificity, and the analysis time was <1 h [77]. The same configuration was used for the detection of the three most common human papillomavirus types in the same test line, while a second line was used to detect a control housekeeping gene, that of b-actin. This strip had an LOD of 100 and 10 copies/reaction for HPV16, HPV18, and HPV45 types and 1 ng/reaction for gDNA after a 30 min reaction [78]. A similar configuration was used for the development of a LAMP strip test for the simultaneous detection of the bacteria *Raillietina* spp. and *Ascaridia galli* in chicken with an LOD of 5 pg/μL within 70 min. The difference lay in the use of FAM-labeled specific probes. The specific hybridization of the amplified products to these probes led to a reduction in false-positive results (Figure 6B) [79]. A similar approach exploited LAMP for the amplification of *Salmonella* and *Shigella* bacteria. The amplicons were labeled with biotin and Dig or FITC and detected by a dual strip by SA–AuNPs. This method was completed in 80 min and had an LOD of 3.4–3.9 CFU/reaction for both targets. The strip showed also excellent specificity [80]. A dual LAMP-LFA was also developed for the simultaneous detection of the bacteria *Salmonella* and *Staphylococcus* based on the genes *Salmonella* enterotoxin and femA. Single-stranded biotinylated LAMP products were produced. The products were captured on the strip by immobilized specific probes, instead of antibodies, and visualized by anti-biotin Ab–AuNPs conjugates forming red spots. The system was applied to drinking water and eggshells, with a limit of detection of 1.6 CFU of *Salmonella* and down to 9 aM (~5.44 copies/mL) of *Salmonella* genomic DNA with high specificity among other bacteria (*Klebsiella*, *Pseudomonas*, *Enterobacter*, *Staphylococcus*, and *Escherichia coli*). Moreover, the novel design of this approach reduced false-positive signals on the strip due to non-specific amplification [81].

### 3.3. Recombinase Polymerase Isothermal Amplification (RPA)

RPA is another isothermal amplification technique that is based on the activity of three essential proteins: a recombinase, a single-stranded DNA binding protein (SSB), and a DNA or RNA polymerase. During RPA, a nucleoprotein is formed by the combination of the recombinase with each primer that leads to subsequent binding to the complementary DNA target. SSB ensures that the two DNA strands do not hybridize back together. Subsequently, the DNA polymerase that has also strand displacement activity synthesizes the new DNA strands. By incorporating an RNA polymerase into the RPA system, reverse transcription can also be performed, enabling the direct analysis of RNA sequences. Compared with LAMP, RPA uses only two primers instead of four to six and requires lower temperatures for reaction and even less analysis time (<15 min). Therefore, RPA is a more simple and user-friendly approach. In addition, the integration of RPA with LFA has dramatically enabled field testing [82,83,84].

In a specific approach, three viral pathogens, bovine viral diarrhea virus (BVDV), bovine epidemic fever virus (BEFV), and bovine respiratory syncytial virus (BRSV), were simultaneously detected in cattle by a multiplex RT-RPA and LFA system. RPA products were labeled for the three pathogens with biotin at one end and Dig, FAM, or ROX at the other end. The products were captured at the test zones of the LFA via anti-Dig, anti-FAM, and anti-ROX antibodies and visualized by anti-biotin Ab–AuNPs. The total assay was completed within 33 min and had LODs of 262, 242, and 256 copies/μL for BVDV, BEFV, and BRSV, respectively. The assay showed no cross-reactivity with six other pathogens and excellent intra- and inter-assay precision with CVs of 2.9–4.0%. Also, the diagnostic sensitivity and specificity were 98.11% and 100%, respectively, indicating only a small chance of false negatives [82]. Similarly, three foodborne bacteria, namely, *Staphylococcus aureus*, *Vibrio parahaemolyticus*, and *Salmonella Enteritidis,* were detected by a triplex RPA-LFA. Herein, the RPA was labeled with Dig at one end and Cy5, FAM, and biotin at the other end. The whole procedure was completed within 15 min with LODs of 26, 76, and 12.9 CFU/mL for the three pathogens. The method also demonstrated good recoveries, 92–108% in spiked food samples, proving the method‘s effectiveness [84].

Moreover, CRISPR and CRISPR-associated (Cas) enzymes have emerged as novel and valuable diagnostic tools to improve the specificity and detectability of nucleic acid analytical methods. The Cas enzymes specifically recognize and cleave amplified DNA products that are complementary to the CRISPR RNA (crRNA), minimizing false-positive results caused by primers, dimers, or non-specific amplicons. After the activation of the Cas enzymes by the target, the Cas enzymes cleave the reporter probes that induce signal amplification and thus increase detectability. Multiplex CRISPR reactions are highly needed. In this work, the on-site detection of African swine fever virus variants was accomplished through RPA in combination with a CRISPR-Cas12b/Cas13a assay. Two genes (B646L and MGF505-2R) were amplified during a multiplex RPA. The T7 RNA polymerase promoter was added to the 5′ end of the forward primer of B646L to allow subsequent transcription, which also prompted signal amplification. For the subsequent CRISPR-Cas12b/Cas13a detection system, the Cas enzyme recognized specific RPA amplicons that had a complementary sequence to the guide RNA, thereby eliminating the false-positive signals derived from non-specific amplicons or primer dimers. Then, the activated Cas enzyme cleaved single-strand DNA (ssDNA) or single-strand RNA (ssRNA) sequences, resulting in the production of a fluorescent or color line on a lateral flow strip. The Cas13a enzyme, a crRNA guide RNase, bound to B646L transcripts that were generated by the in vitro transcription of the T7 promoter-DNA RPA products, resulting in cleavage of ssRNA probes but not the ssDNA probes. In contrast, the Cas12b enzyme, a single guide RNA (sgRNA) that guides DNase, recognized the MGF RPA products and led to cleavage of ssDNA probes but not ssRNA probes. The cleaved products were detected by a commercially available double test strip (HybriDetect 2T, Milenia) by immobilized SA and anti-Dig Abs and anti-FAM-AuNPs as reporters. The whole assay was completed within 60 min and had LODs of 1.6 copies/μL and 8 copies/μL for the two genes [85]. The HybriDetect lateral flow strip was also used for the detection of the SARS-CoV-2 virus in less than 25 min. The nucleocapsid protein gene of SARS-CoV-2 was simultaneously detected with the gene of the MS2 bacteriophage as a positive control lysis, after reverse transcription and amplification through RPA. The RPA products were labeled as above. The LOD of this method was 50 copies/reaction [83]. Again, a CRISPR-Cas12b/Cas13a-based LFA was developed for the detection of genetically modified (GM) crops. A dual RPA-CRISPR-Cas12b/Cas13a reaction was performed in the same tube for the simultaneous detection of the Cauliflower Mosaic Virus 35S (CaMV35S) promoter and the nopaline synthase terminator (NOS) terminator for GM identification using a commercially available dual strip (Tiosbio, CRISPR Double Enzyme Cutting Nucleic Acid Detection Strip). The CaMV35S gene was selected as the target of Cas13a and the NOS gene as the target of Cas12a during the CRISPR detection dual system. As previously described, the Cas13a enzyme recognized only RNA; thus, the forward primer for CaMV35S carried a T7 promoter for subsequent transcription to ssRNA to activate Cas13a. Additionally, a protospacer adjacent motif (PAM) was necessary for Cas12a activation, and for that reason, NOS products carried PAM. These modifications ensured that both the Cas13a and Cas12a enzymes would bind to their respective crRNAs, with no cross-reactivity between the two systems. For the detection with the dual LFA, the ssDNA reporter probes were labeled with fluorescein isothiocyanate (FITC) and biotin, while the ssRNA reporters carried a Dig and biotin moiety at both ends. For the visual detection, anti-mouse FITC-AuNPs and rabbit anti-Dig AuNPs were used, while anti-rabbit nanoantibody and goat anti-mouse secondary Ab were immobilized at test lines 1 and 2, respectively. Finally, down to two copies were detectable for both targets within 35 min in one pot, eliminating contamination [86]. The same strategy was also exploited for the development of a dual strip for the simultaneous detection of two human papillomaviruses (HPVs), HPV16 and HPV18. The assay was completed in 40 min and had an LOD of 10 copies/μL [87]. Also, Wang et al. in 2025 utilized CRISPR/Cas12a and RPA for detecting GM crop markers with high specificity, achieving detection limits of 10 copies/µL in less than 50 min without complex equipment [88].

A dual-color strip was developed for the simultaneous detection of chicken and duck adulteration in mutton. Two colored AuNPs, gold nanospheres (red) and gold nanoflowers (blue), were synthesized and conjugated to DNA probes complementary to each DNA target for easier interpretation of the colored lines on the same strip. RPA products were labeled at both ends with specific tags that were captured by complementary anti-tag sequences at both test lines of the strip. The hybrids were finally detected by AuNP conjugates. This method was based only on hybridization between DNA sequences, avoiding the use of antibodies, lowering the cost of the analysis, and enhancing the multiplexing potential. The method showed an LOD of 0.01% adulteration for both targets and excellent specificity among eight different meat species [89]. In another approach, again for meat adulteration detection, the pork (*Porcine*) mitochondrial ND2 gene and the chicken (*Gallus gallus*) cytochrome B gene were amplified by a dual RPA. A novel DNA isolation device, an “extraction stick”, a stainless-steel stick that consisted of a nitrocellulose membrane coated with mesoporous UiO-66 (zirconium 1,4-icarboxybenzene metal organic framework)-NH_2_, was fabricated for the rapid isolation of DNA within 1 min. The amplified products were also labeled with biotin and either Dig or FAM in this approach. The detection was accomplished by SA-AuNPs conjugates in 18.5 min and with an LOD of 0.1% of adulteration. The strip showed excellent specificity among seven meat species [90]. Finally, a triplex visual detection of tobacco potyviruses combined with RT-RPA was also carried out. The LFA was used for the simultaneous detection of coat protein (CP) genes of potyviruses potato Y virus, chilli veinal mottle virus, and tobacco vein banding mosaic virus. The RPA products were labeled at one end with Dig, FAM, and TAMRA and at the other end with biotin. They were detected with the triplex strip by immobilized anti-Dig, anti-FAM, and anti-TAMRA monoclonal Abs and SA-coated AuNPs as reporters. The LFA provided an LOD of 10^3^ copies per reaction within 35 min with no cross-reactivity between the different viruses [91].

### 3.4. Other LFAs

A dual strip was developed for the simultaneous detection of two micro-RNAs (miRNAs). Two molecular beacons (MBs) that contained a complementary segment for each of the two miRNAs tested, miR-210 and miR-424, were immobilized at test lines 1 and 2, respectively. AuNPs were conjugated to DNA probes that were complementary to another fragment of the MBs. Upon the addition of the miRNA targets, the miRNAs opened the MBs, and a sandwich complex with the AuNP conjugates was shaped forming two red test lines. This dual strip had an LOD of 10 pmol and good selectivity among one- to five-base mismatched miRNAs; however, no real-sample analysis was reported [92]. A sandwich-type nucleic acid hybridization reaction-based LFA was developed for the detection of three miRNAs, miR-21, miR-155, and miR-210. AuNPs were also conjugated to DNA probes that were complementary to a segment of the miRNAs, while miRNA targets were captured through immobilized recognition oligonucleotides that were complementary to the rest of the micro-RNA sequences. The detection limits of this LFA were estimated to be 0.007 nM, 0.068 nM, and 0.017 nM for the three miRNAs. The specificity was very good for two-base mismatched miRNA sequences and random miRNA, but the proposed LFA could not distinguish between one-base mismatched miRNA [4]. A similar approach was developed for the simultaneous detection of three synthetic DNA sequences that correspond to the H1N1, H3N2, and H9N2 influenza viruses. Fe_3_O_4_–Au nanoparticles were used here as reporters, as alternative materials to enhance detectability. The nanoparticles were coupled to SA, and three sets of conjugates that were complementary to the three targets were produced with biotinylated DNA detection probes. A set of three other DNA recognition probes, which were also biotinylated, were used to form the three test lines of the strip through SA-assisted immobilization. This multiplex LFA showed LODs of 0.5–1 nM of synthetic targets, with good specificity and reproducibility indicated by CV values <12%. These high detection limits in the nanomolar level, however, may be attributed to the fact that no nucleic acid amplification technique was used [93].

The simultaneous detection of the pathogens *Staphylococcus aureus*, *Listeria monocytogenes*, and *Salmonella typhimurium* was also carried out through hybridization chain reaction (HCR), subsequent treatment with the enzyme exonuclease III (exo III) and a multiplex LFA. This method targeted the bacterial 16S rRNA sequence, and CdTe/CdS QDs were used as reporters for fluorescent signal production. In more detail, two hairpins were used for each target. The target opened the first hairpin that triggered the HCR reaction between the two hairpins. The first hairpin carried also an ssDNA probe that was the target for exo III. HCR produced multiple ssDNAs that hybridized then to a third hairpin. Upon the addition of exo III, ssDNAs were released along with a single-stranded sequence from the third hairpin. The lateral fragments were captured on the strip by immobilized target recognition probes and CdTe/CdS QDs coupled to complementary DNA probes to the other site of the single-stranded fragments. This system provided LODs of 47, 43, and 56 CFU/mL for *Staphylococcus aureus*, *Listeria monocytogenes*, and *Salmonella typhimurium*, respectively, after total RNA isolation, while recoveries for the three targets in milk samples were 97.5−107.0, 98.7−106.6, and 99.4−108.3%, respectively, and with CVs ranging from 1.2 to 5.1%. Finally, a portable fluorescence detection device was constructed consisting of a 3D-printed card bracket, a support slot, and a miniaturized fluorometer to allow for quantitative analysis [94]. SERS was also combined with a multiplex LFA and the amplification technique of catalytic hairpin assembly (CHA) for the simultaneous detection of two micro-RNAs, miR-106b and miR-196b, as laryngeal squamous cell carcinoma biomarkers in human serum. SERS–LFAs have been introduced for ultrasensitive detection of various analytes. Among other amplification techniques, CHA can be performed at room temperature and provides lower background signals, being a promising tool for sensitive detection of nucleic acids. Palladium gold nanorods (Pd–AuNRs) were used here as reporters that were coupled to two hairpins complementary to the two miRNA targets, carrying a biotin moiety at one end. The core-shell formation and the introduction of Pd–AuNRs, instead of AuNRs, resulted in an enhanced SERS signal. To the one set of Pd–AuNRs, mercaptobenzoic acid was coupled, while the other set of nanoparticles were conjugated to Nile blue A to construct two Raman reporters. The Pd–AuNRs conjugates were applied onto the conjugate pad of the strip along with miRNAs. The targets opened the corresponding hairpin probes, and subsequently, the hybrids were captured at the test lines where CHA began through hybridization to immobilized complementary hairpins. Finally, immobilized SA captured the biotinylated strands. The accumulation of SERS tags formed gray lines, while the measured SERS signals enabled quantitative analysis. The detection limits for miR-106b and miR-196b were calculated to be as low as 23.17 and 46.94 aM in buffer and 43.08 and 61.36 aM, respectively, in human serum. The whole detection process was completed within 1 h [95]. As a last approach, a hybridization assay, utilizing a nucleic acid-based lateral flow platform and SERS, was developed for meat adulteration detection. The SERS-based LFA incorporated a DNA amplification step utilizing RPA. The amplified DNA products were simultaneously detected on the test lines of the LFA strip using SERS tags. These tags consisted of complementary DNA probes specific to the amplified sequences, conjugated with red and blue AuNPs, enabling dual colorimetric and distinct SERS signal generation. The assay was successfully applied to real meat samples, achieving a detection limit of 0.01% adulteration [96].

## 4. Aptamer-Based Lateral Flow Assays

Aptamers are ssDNA or RNA and have been used as alternatives to antibodies to lower the cost and increase the specificity and flexibility/versatility of the tests. LFAs have exploited aptamers for the detection of various targets in different LFA formats. The conformational diversity of aptamers was responsible for recognizing a wider variety of targets, such as amino acids, metal ions, polysaccharides, proteins, virus, bacteria, and whole cells, through hydrogen bonding, electrostatic interactions, shape effect, and π–π interactions. They also provide high flexibility and versatility because new aptamers can be easily synthesized with low cost [55]. In one approach, multicolored UCNPs were used as reporters for the development of a multiplex LFA for the simultaneous detection of three different kinds of targets: mercury ions, ochratoxin A, and *Salmonella* bacteria. The LFA was integrated with a smartphone for visual detection of emitted fluorescence. Three kinds of UCNPs (red, green, and blue) were conjugated to different aptamers specific to three targets. An LFA in a competitive format was then constructed. In the case of a negative sample, the UCNPs conjugates were hybridized to immobilized complementary DNA probes forming three colored test lines of different fluorescence colors. The excess of nanoparticles was captured by a complementary DNA probe for the three aptamers at the control line of the strip. In the presence of the targets in the sample, the conjugated nanoparticles bound to the targets, and no test line was formed. The excess of nanoparticles again traveled through the strip forming only the control line. The LFA was completed in 30 min and provided LODs of 5 ppb, 3 ng/mL, and 85 CFU/mL for mercury ions, ochratoxin A, and *Salmonella*, respectively, with good specificity [55]. A three-channel aptamer-based lateral flow assay (Apt-LFA) was also established for the simultaneous detection of aflatoxin M1(AFM1), aflatoxin B1 (AFB1), and ochratoxin A (OTA), avoiding cross-reactions compared with a single multiplex strip. Each strip consisted of one test line and was prepared separately. The three strips were glued together on a backing plate. Gold-iridium nanoparticles (Au@Ir NPs) were synthesized and coupled to polyA complementary (cDNA) probes through an Au–S bond (Au@Ir@SH-Poly A-cDNA) as an attempt to increase the sensitivity of LFAs that commonly use AuNPs as reporters. Among other amplification strategies, nanozymes have attracted much attention. Nanozymes catalyze colorimetric substrates to produce multiple color signals enhancing the analytical performance of LFAs. Compared with the common colorimetric signal of AuNPs, nanozymes can increase the signal by several orders of magnitude. In this work, in the presence of the targets in the sample, the aptamers were incubated with the sample and then loaded to the strip. Each target was bound to its corresponding aptamer. The unbound nanoparticles were then captured to the test line by immobilized DNA probes. By adding a chromogenic substrate, the nanoparticles catalyzed the formation of an intense blue color at the test line. In the absence of the targets, the aptamers were free to bind to the complementary probes at the test line blocking the formation of the blue color. This LFA was rapid and showed detection limits of 0.39 ng/mL, 0.36 ng/mL, and 0.82 ng/mL for AFM1, AFB1, and OTA, respectively. Also, the test after the real sample analysis of complex matrices provided recoveries of 93.33–97.01%, 95.72–102.67%, and 106.88–109.33% for the three targets. Compared with AuNPs, the catalytic formation of the blue product on the test line enhanced the detectability of the proposed LFA [97].

## 5. LFAs for the Simultaneous Detection of Different Biomolecule Categories

**Figure 7:**
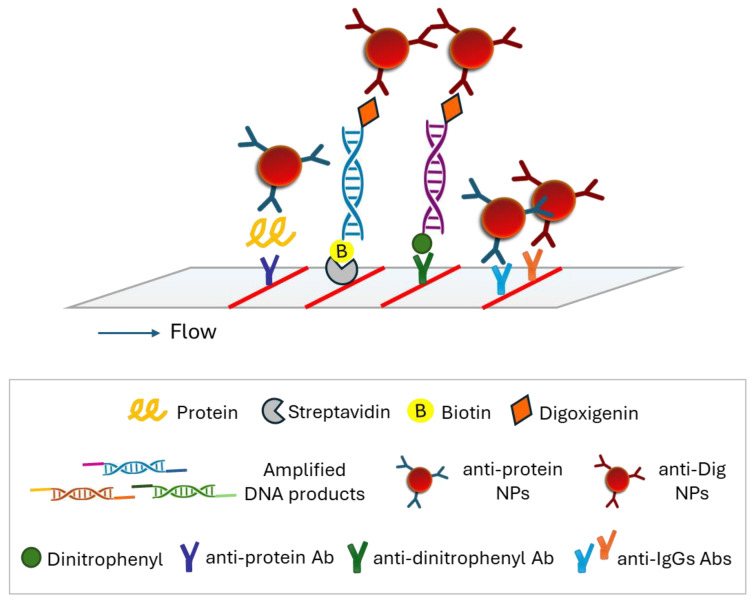
A lateral flow assay for the simultaneous detection of different categories of biomolecules, such as proteins and nucleic acids. Amplified DNA products were labeled at both ends with antigens (biotin, dinitrophenyl, and digoxigenin (Dig)). Proteins and DNA products were captured at the multiplex LFA through specific antibodies (Ab) and streptavidin and detected by different Ab-coupled nanoparticles (NPs) [99].

The diagnosis of complex diseases and the evaluation of treatment responses are typically linked to multiple biomolecules rather than relying on a single identifiable biomarker. As a result, gaining insights into disease progression or therapeutic effectiveness necessitates the analysis of a range of biologically relevant biomarkers [98]. In this context, the development of multiplexing technologies has significantly improved the efficiency of such analytes. These technologies allow for the simultaneous measurement of different biomolecules from a single sample under uniform conditions. A perfect proposal toward this new direction is the development of a multianalyte LFIA for the simultaneous detection of *Pseudomonas auruginosa* gDNA and the protein interleukin-6 (IL-6) in wound exudate. The assay operated under the innovative concept of “nucleic acid-to-protein transformation”, where both analytes were added directly into RPA reaction, in order to amplify the target DNA at a rapid pace. During the amplification process, specific primers and probes were utilized to introduce antigenic labels onto the resulting double-labeled target DNA amplicons. Next, the reaction mixture was diluted and applied to the strip, and sandwich immunoassay took place. In this step, the labeled amplicons and the target protein were captured by specific antibodies immobilized on the test lines of the strip. The detection was facilitated by the use of fluorescently labeled microspheres, bound to the captured analyte, allowing for quantification through fluorescence imaging (Figure 7). The entire assay could be completed in 35 min, making the proposed LFIA a rapid and effective tool for POC diagnostics, while it showed enhanced specificity and sensitivity, with limits of detection reaching 4 ng/mL IL-6 and 70 copies/reaction of *P. aeruginosa* genomic DNA [99].

## 6. Discussion

LFAs are paper-based simple colorimetric tests that have been employed for rapid detection of various targets at points of care and in the field. LFAs are designed in a very simple way to be utilized by non-specialized individuals. LFAs are easy to use, rapid, portable, and cost-effective, and in general, small volumes of samples and reagents are required. The combination of LFAs with amplification techniques is also very attractive. LFAs have been applied for analysis in many different scientific fields from clinical diagnostics to food and environmental testing. The COVID-19 pandemic has just proven their usefulness for mass usage. As multianalyte testing is the gold standard in (bio)analytical sciences, to get as much information as possible within a short period of time, multiplex methods and devices are in great demand. LFAs are classified in two main categories: LFIAs and nucleic acid-based LFAs. LFIAs have the great advantages of fast analysis and direct analysis of the sample usually without or with minimal pre-treatment. On the other hand, nucleic acid-based ones provided lower detection limits and greater specificity, especially among closely related species.

The use of nanoparticles/nanomaterials, as well, provided signal enhancement and increased the detectability of such tests. The exploitation of novel fluorescent and luminescent nanoparticles with narrow emission peaks and wide emission range increased the multiplicity compared with common fluorescent dyes that limit multiple detection due to overlapping of emission spectrums. Gold nanoparticles are the most frequently used reporters that generate colorimetric signals. Different particles, such as colored latex microparticles, enabled multiplex discrimination compared with gold nanoparticles and provided easier bioconjugation and more stable conjugates. Novel nanoparticles or nanomaterials have emerged in many applications as an attempt to increase the detectability and the specificity of LFAs. By integrating new nanostructures, such as proteinticles as 3D probes in LFIAs, 100% diagnostic sensitivity was achieved. Similarly, dual-mode nanoparticles, combining colorimetric and fluorescence properties, have emerged as powerful tools to enhance both the sensitivity and specificity of the LFAs, particularly in complex sample matrices.

As mentioned before, some major drawbacks of LFAs include the low detectability and in some cases the low specificity. The introduction of novel nanomaterials and sophisticated configurations in combination with signal enhancement techniques has contributed to signal increasement. The specificity of LFAs is an issue that has to be addressed. Simulation techniques may play a key role in the selection of the optimum biorecognition molecules, while optimization of all steps could enhance the specificity of interactions. Limitations of LFIAs are often the detectability, as no techniques for amplification of the protein content exist, as well as the specificity. Therefore, DNA-based ones are usually the preferred analytical tools due to DNA stability and because they combine the power of amplification of the targets. For bacteria and pathogen detection, culture methods require a minimum of three days for a positive result, whereas more days are needed for a negative result. Accurate and in-time bacteria and virus detection is needed to prevent rapid spread of the pathogens among individuals or in the environment. LFAs compose a powerful tool for rapid and early pathogens detections. LFAs, especially the antigen-based ones, have dramatically reduced the time of the analysis. Moreover, multiplex LFAs are in high demand for detecting multiple pathogens in a single sample on one strip, thereby reducing both analysis time and costs.

To perform DNA-based LFAs, capture DNA or RNA probes are used as recognition elements of the targets. The streptavidin–biotin interactions are mostly used as the detection system of LFAs, or streptavidin is immobilized at the test zone to bind the DNA capture probes that are biotinylated for this purpose. In addition, other molecules/haptens, e.g., digoxigenin or FAM/FITC and their specific antibodies, e.g., anti-digoxigenin or anti-FITC antibodies, are required for the generation of colorimetric signals. As an alternative, unmodified DNA probes are directly immobilized on the nitrocellulose membrane. The direct immobilization of captured DNA oligonucleotides is highly advantageous as it reduces the required labels for signal generation and thus the overall cost of the LFAs. DNA-based LFAs provide also detection and simultaneous identification of specific DNA sequences.

Isothermal amplification techniques, moreover, have gained wide attention because they perform nucleic acids amplification at constant temperature without the need of special instruments, enabling on site detection and reducing the time and the temperature of the amplification reaction. However, particularly for LAMP reactions, the complex design of multiple primers for each target and the need for expensive enzymes limit their applicability for multiplex testing. In those cases, common PCR may be the gold standard technique, especially when combined with simple and cost-effective post-PCR detection techniques with high multiplexing potential, such as LFAs. The specificity of amplification reactions relies on the target sequences, the primer sequences, and the size of the amplicons. Therefore, reliable software for designing highly specific primers, especially for multiplex isothermal amplification techniques, is currently required. In addition, a weakness of multiplex CRISPR reactions is the lower enzyme activity upon combination of different enzymes that has to be carefully optimized.

Moreover, degradation of nucleic acids, especially in highly processed samples, affects the detectability of nucleic acid-based LFAs. To overcome this problem, short sequences of degraded DNA should be targeted. DNA barcoding has evolved toward this direction enhancing the detection and the specificity of close species discrimination. Moreover, DNA isolation is a crucial step for the successful implementation of a method; therefore, this step must be well optimized, and novel techniques that will enhance efficiency and reduce the processing time are strongly required. Another issue is that DNA-based methods cannot distinguish between live and dead cells in the case of infection detection. Filtration may solve this problem, wherein it enriches the sample, which leads to increased detectability. Filtration may also remove possible PCR inhibitors that are responsible for lower amplification efficiency. Moreover, the dually labeled, with haptens, amplified products that are detected with an LFA have increased probability for false-positive signals. The use of two haptens also increases the cost of the analysis. Hybridization with specific DNA probes and direct immobilization of DNA recognition probes on the membrane of the strip may be a useful alternative to deal with these drawbacks.

Most of the existing multiplex LFAs, both immunoassays and nucleic acid-based ones, however, have limited their applications to the detection of two to three analytes. This is attributed, probably, to lower detectability and specificity in the case of multiple targets, with the specificity being the most significant factor. There are only a few reports that were able to detect up to four analytes, such as using four antibodies that recognized portions of peptin structure [9], tetracyclines [54], viruses [46,47], drug residues [41], and veterinary drugs using [26]. As reporters in the above reports, dual-emission fluorescent CDs@Eu^3+^, magnetic nanoparticles, AuNPs, gold nano-urchins, quantum dot nanobeads, quantum dots, and SERS nanotags were used. Only in one report were the authors able to detect five mycotoxins in a single strip using AuNPs and time-resolved fluorescence microspheres conjugated with antibodies for visual signal production [53]. Increased multiplicity ranging from two to five SNPs, thus 4–10 alleles, was only achieved for the detection of specific SNPs targeting various genes related to several diseases [66,67,68,69,70,71,72,73]. Finally, a multispecies DNA sensor-LFA was developed for the identification of seven edible oils for olive oil adulteration detection based on different SNPs present in each plant’s genome [74] and a multi-allelic sensor that was able to detect two SNPs, thus four alleles, in a single strip for the identification of the olive oil’s varietal origin [75]. All the above multiplex LFAs for SNP detection used AuNPs for visual detection. Moreover, among all approaches, the combination of LFAs with SERS, and common amplification techniques, e.g., CHA for nucleic acids, provided the lowest detection limits. For nucleic acid-based LFAs, the one with combination with isothermal target amplification techniques, especially RPA, or other amplification systems, such as HCR and CHA, resulted in much faster analysis and lower detection limits. As for protein analysis, the most sensitive LFIAs were the fluorescent and the SERS-based, achieving detection limits on the level of fg-pg/mL. Table 1 presents an overview of all reports for multiplex LFAs regarding principle, signal output, reporters, and multiplicity.

To increase the applicability of the DNA/LFA-based methods, an extraction-free sample must be able to be analyzed in a highly integrated system for amplification reaction and LFA. Thus, faster analysis would be achieved, and an integrated system would eliminate errors induced by the users. Despite the growing demand for advancements in POC devices, only 9% of the reports integrate smartphone integration, highlighting a missed opportunity for innovation. Smartphones offer significant advantages, including real-time data analysis, enhanced diagnostic sensitivity, and remote sharing capabilities. The possibility of combining algorithms and artificial intelligence can equip POC tools with advanced features. Therefore, greater efforts to integrate smartphones into diagnostic platforms are crucial for the future of decentralized healthcare solutions. To enhance their usefulness, however, automative image analysis tools and/or artificial intelligence tools with applications to LFAs are urgently required for fast and reliable results, avoiding subjective interpretation of the multi-line results or patterns obtained from multiplex LFAs.

Among these approaches, there are only a few types of Food and Drug Administration (FDA)-approved assay principles for very specific targets. A recent review of the FDA-approved in vitro diagnostic tests database (https://www.accessdata.fda.gov/scripts/cdrh/cfdocs/cfIVD/Search.cfm (accessed on 17 January 2025)), encompassing both at-home and healthcare-provider-performed tests, reveals over 500 approved devices, most of them being for single-analyte detection. These entries focus on the detection of *(i)* COVID-19 and other respiratory diseases, such as influenza A and B, using antigen-based assay principles; *(ii)* multi-drug urine tests, which also employ antigen-like principles specific to each target; and *(iii)* leukocyte and nitrite detection in urine. However, approved tests targeting *Streptococcus pyogenes* Group A and microorganisms associated with vaginitis and bacterial vaginosis, utilizing non-invasive swab sample collection, use PCR amplification methods rather than lateral flow approaches.

This highlights a gap in the availability of rapid, multiplex LFAs capable of addressing a wider range of targets without requiring further amplification. Furthermore, if amplification is necessary, it underscores the need for more robust lateral flow systems capable of detecting PCR products or achieving higher levels of multiplexity to meet clinical demands. The FDA approval landscape reflects that current LFA technology is not yet sufficiently advanced for widespread clinical application, particularly for complex multiplex detection. Researchers should adopt a more translational approach, focusing on developing and optimizing technologies that can be readily adapted to clinical devices for real-world use.

## 7. Conclusions

In conclusion, multiplex lateral flow assays combined with amplification strategies are required for multianalyte biomolecular analysis in various fields. Multiplex immunoassays account for great simplicity and rapid analysis, making them the most promising candidates for POC testing and rapid field detection, while nucleic acid-based LFAs have a positive effect on the detectability and specificity of the methods.

Multiplex LFAs offer several advantages over traditional methods like ELISA or PCR, but they also have limitations. One major advantage is the rapid result time, typically within minutes to hours, much faster than the hours or days required for PCR and ELISA. LFAs are also user-friendly, as demonstrated by FDA-approved at-home tests, whereas traditional methods often require laboratory infrastructure and trained personnel. They are cost-effective and portable, enabling on-site testing with minimal equipment, and generally have lower costs compared with traditional ones, which involve expensive reagents and specialized equipment. Additionally, multiplex LFAs require minimal reagents and sample volumes, often using bodily fluids such as blood or urine.

At the moment, the multiplicity of the existing LFAs is still limited, which often compromises their sensitivity and specificity. They generally exhibit lower sensitivity and specificity than traditional methods, particularly for low-abundance targets, which can lead to false positives or negatives. LFAs are usually qualitative or semi-quantitative, offering “yes/no” results, rather than precise measurements, limiting their use in applications that require accurate quantification. Increased multiplicity, however, succeeded in methods that aimed for the detection of various SNPs in one sample. Therefore, the number of analytes detected by multiplex LFAs must be increased in order to enhance the applicability of LFAs in biomolecular rapid diagnostics. Efforts toward this direction may include the exploitation of new labels or the production of new antibodies and aptamers for increased specificity in a multiplex LFIA. As for nucleic acid-based LFAs, in order to increase multiplicity and overcome specificity issues, careful design of the recognition probes using reliable software must be carried out. To this context, several novel nanoparticles and nanomaterials, as well as sophisticated signal amplification techniques, have been integrated with LFAs aiming to increase the multiplexing potential, the detectability, and the specificity of these tests. In order to make multiplex LFAs more suitable for on-site testing, fully integrated devices are required. Moreover, in order for multianalyte LFAs to account for all users, automative systems based on smartphones and image analysis or artificial intelligence tools for automatic interpretation of the multiple visual signals must be developed, avoiding misinterpretation and allowing also quantitative analysis. To conclude, while multiplexing offers the potential for broader diagnostic applications, achieving a balance between the number of analytes and maintaining high sensitivity is crucial. There is a trade-off, especially for low-concentration analytes, where increasing plex may compromise performance. At this point, as already discussed, it should be highlighted that AI approaches and chemometric methods, combined with experimental design, can provide the necessary balance.

Finally, future perspectives will account for the development of combined LFAs that could simultaneously detect various biomolecules of different categories, such as the simultaneous detection of multiple proteins and DNA/RNA sequences.

## Figures and Tables

**Table 1:** An overview of the reports regarding the analytical performance of multiplex LFAs.

Signal	Target	Method	Reporters	LOD	Multiplicity	Ref
**Lateral flow immunoassays**						
***Simple colorimetric***						
Color	PJI proteins	Sandwich immunoassay	AuNPs–antibody	-	2	[7]
Color	Mycotoxins	Sandwich immunoassay	AuNPs–antibody	0.10–0.46 μg/kg	**3**	[8]
Color	Pectin structures	Sandwich immunoassay	Gold NPs and gold nano-urchins	0.02 μg/mL	**4**	[9]
Color	Virus	Sandwich immunoassay	AuNPs–antibody	-	**3**	[10]
Color	Fungicide	Competitive immunoassay	AuNPs–antibody	2.0–8.8 ng/g,	**3**	[11]
Color	Virus	Sandwich immunoassay	AuNPs–antibody	2.44–4.88 ng/mL	2	[12]
Color	Virus	Sandwich immunoassay	AuNPs–antibody	-	**3**	[13]
Color	Steroid hormones	Sandwich immunoassay	AuNPs–antibody	0.32–0.64 μg/kg	**3**	[14]
Color	Virus	Sandwich immunoassay	AuNPs–antibody	2.9–6.3 CFU	**3**	[15]
Color	Intercellular adhesion molecule 1 (ICAM1), carbohydrate antigen 19-9 (CA19-9), and prostate-specific antigen (PSA)	Sandwich immunoassay	Plasmon-controlled metal–silica isoform nanocomposites	6.65 ng/mL, 0.04 U/mL, and 0.12 ng/mL	**3**	[16]
Color	Pollutants	Sandwich immunoassay	AuNPs–antibody	0.67–2.22 ng/mL	2	[17]
Color	Toxins	Competitive immunoassay	AuNPs–antibody	10–30 ng/mL	**3**	[18]
Color	Bacteria	Sandwich immunoassay	AuNPs (Cu_2_ deposition)	0.01–0.03 μg/mL	2	[19]
Color	Metabolites	Cascaded antibody directionality (CAbD)	Ab-ZnO-based nanocomposites	0.060–0.106 ng/mL	2	[20]
Color	Proteins	Sandwich immunoassay	Polystyrene microspheres	2.5–7.8 ng/mL	**3**	[21]
Color	Allergens	Sandwich immunoassay	Metal nanoparticles	1 mg/L–1%	**3**	[22]
***SERS***						
SERS	Toxins	Immunoassay	SERS nanoprobes–antibodies	0.11–15.7 pg/mL	**3**	[23]
SERS	Cardiac biomarkers	Sandwich immunoassay	Core-shell SERS nanotags	0.44–3.2 pg/mL	**3**	[24]
SERS	Serum amyloid A/C-reactive protein	Immunoassay	Fe_3_O_4_@Au nanotags–antibodies	0.1 ng/mL and 0.01 ng/mL	2	[25]
SERS	Kanamycin/ractopamine/Clenbuterol/chloramphenicol	Competitive immunoassay	Magnetic-core dual-shell nanoparticles–antibodies	0.52, 2.5, 0.87, and 6.2 pg/mL	**4**	[26]
SERS	Chlorothalonil/imidacloprid/oxyfluorfen	Immunoassay	Ag@AuNPs nanotags–antibodies	-	**3**	[27]
SERS/Thermal Signal	Influenza A/influenza B/SARS-CoV-2	Immunoassay	Gold-core–silver-shell bimetallic nanoparticles–antibodies	62.5, 750, and 31.25 pg/mL	**3**	[28]
SERS	Carbendazim/imidacloprid	Immunoassay	Dual-SERS nanotags–antibodies	0.04 and 0.06 ng/mL	2	[29]
SERS	Troponin I/N-terminal natriuretic peptide precursor	Immunoassay	AuNPs–polystyrene tags–antibodies	1 and 10 pg/mL	2	[30]
SERS	Alzheimer’s biomarkers	Immunoassay	Gold–silica nanotags–antibodies	~16 fg/mL	2	[31]
***Fluorescent***						
Fluorescence	Mycotoxins	Competitive immunoassay	Ab–SiO_2_@QDs	50–500 μg/kg	2	[32]
Fluorescence	Proteins	Sandwich immunoassay	Ab–dual color QDs	0.29–0.66 ng/mL	2	[33]
Fluorescence	Proteins	Competitive immunoassay	Ab–SiO_2_@QDs	0.007–0.32 ng/mL	2	[34]
Fluorescence	Proteins	Sandwich immonoassay	Ab–QDs	0.05–0.5 μg/mL	**3**	[35]
Fluorescence	Antigens	Sandwich immunoassay	Ab–DMSNs–BCDs	0.01 ng/mL and 0.1 U/mL	**3**	[36]
Fluorescence	Proteins	Sandwich immunoassay	SA–Au nanoclusters	0.003–0.023 mg/L	2	[37]
Fluorescence	Toxins	Sandwich immunoassay	Ab–magnetic QD NPs	2.52–2.86 pg/mL	2	[38]
Fluorescence	Antigens	Sandwich immunoassay	SA–SiNPs	5 U/mL–5 pM	2	[39]
Fluorescence	Viruses	Sandwich immunoassay	Ab–EuNPs	3.4 × 10^2^–2.1 × 10^4^ TCID_50_/mL	**3**	[40]
Fluorescence and Color	Drug residues	Sandwich immunoassay	Ab–MoS_2_–QDs	0.12–0.36 ng/mL	**4**	[41]
Fluorescence	Mycotoxins	Competitive immunoassay	Aggregation-induced emission nanoparticles	6.12–26 pg/mL	2	[42]
Fluorescence and Color	Drugs	Sandwich immunoassay	Ab–AuNPs/Prussian blue nanoparticles	0.013–0.152 ng/mL	2	[43]
Fluorescence and Color	Pollutants	Competitive immunoassay	Ab–AuNPs and EuNPs	0.003–0.6 ng/mL	**3**	[44]
Fluorescence	Mycotoxins	Inner filter effect and competitive immunoassay	Ab/SA-Au nanoflowers/QDs	0.005–0.4 μg/L	**3**	[45]
Fluorescence	Infectious diseases	Sandwich immunoassay	Quantum dot nanobeads (QBs)	0.11–0.62 NCU/mL	**4**	[46]
Fluorescence	Viruses	Sandwich immunoassay	Ab-QBs	0.01–0.40 ng/mL	**4**	[47]
***Nanozymes***						
Color	RAC/CLE	Competitive immunoassay	Prussian blue nanozymes	0.20 and 0.12 ng/mL	2	[48]
Color	RAC/CLE	Competitive immunoassay	Fe-based tanic acid nanozyme	0.015 and 0.156 ng/mL	2	[49]
Color	Drug residues	Competitive immunoassay	Fe-Au@Pt nanozyme	1.1–10.1 pg/mL	**3**	[50]
***Smartphone-based***						
Color	Snake venoms	Sandwich immunoassay	Carbon NPs–antibody	10–50 ng/mL in spiked plasma and urine, 50–500 ng/mL spiked serum	2	[51]
Color	Deoxynivalenol/zearalenone	Immunoassay	Au@Pt nanozyme–antibody	0.24/0.04 ng/mL	2	[52]
Color/ Fluorescence	Aflatoxins	Immunoassay	AuNPs and time-resolved fluorescence microspheres conjugated with antibodies	0.04–0.75 μg/kg	**5**	[53]
Fluorescence	Tetracyclines	Centrifugated LFA	Dual-emission CDs@Eu^3+^	46.7–72.0 nM	**4**	[54]
Fluorescence	Hg^2+^/ochratoxin A/*Salmonella*	Competitive aptamer-LFA	DNA–UCNPs	5 ppb, 3 ng/mL, and 85 CFU/mL	**3**	[55]
Color	SARS-CoV-2/influenza A/influenza B	Immunochromatografic test	Magnetic nanoparticles–zwitterionic ligands	0.0062, 0.0051, and 0.0147 ng/mL	**3**	[56]
Color	Acute kidney injury biomarkers	Vertical paper-based immunoassay	AuNPs–antibodies	~ 1 ng/mL	**3**	[57]
Fluorescence	Methamphetamine/tramadol	Immunoassay	Silica core quantum dot-shell nanocomposite–antibodies	0.11 and 0.017 ng/mL	2	[58]
**Nucleic acid-based LFA**						
***PCR***						
Color	Shrimps/cytB gene	PCR	Anti-FAM Ab–AuNPs	0.02%–0.01 ng	2	[59]
Color	*Escherichia coli/Salmonella Typhimurium* bacteria	PCR	SA–AuNPs	10–100 CFU/25 ng	2	[60]
Fluorescence	SARS-CoV-2/influenza B virus	PCR	SA–QDs	8.4–14.2 copies/mL	2	[1]
Color	*Salmonella/Vibrio cholerae* bacteria	PCR	Antibiotin–AuNPs	10^4^–10^6^ CFU/mL	2	[61]
Color	*P. carotovorum brasilience/E. coli* O157:H7 bacteria	PCR	SA–AuNPs	1.26–13.7 CFU/mL	2	[62]
Color	SARS-CoV-2	Asymmetric PCR	SA–AuNPs	300 aM	**3**	[2]
Color	Meat species	PCR	DNA–latex microspheres	0.1%	**3**	[63]
Color	SARS-CoV-2 virus	RT-PCR	Antibiotin–AuNPs	1 copy	2	[64]
Color	miR-92a/miR-141/miR-345	Stem-loop RT-PCR	Antibiotin-AuNPs	-	**3**	[65]
Color	KRAS gene	PCR-PEXT	SA–AuNPs	-	**4**	[66]
Color	Toll-like receptor 4 (TLR4) gene	PCR-PEXT	Antibiotin–AuNPs	-	2 SNPs **4 alleles**	[67]
Color	MBL2 gene	PCR-PEXT	Antibiotin–AuNPs	-	2 SNPs **4 alleles**	[68]
Color	HBA1/HBA2 genes	PCR-PEXT	Antibiotin–AuNPs	-	**5 SNPs** **10 alleles**	[69]
Color	ATP7B gene	PCR-PEXT	Antibiotin–AuNPs	-	**5 SNPs** **10 alleles**	[70]
Color	FV Leiden/PTH/MTHFR genes	PCR-PEXT	Antibiotin–AuNPs	-	**3–4 SNPs** **6–8 alleles**	[71]
Color	SIRT1 gene	PCR-PEXT	Antibiotin–AuNPs	-	**4 SNPs** **8 alleles**	[72]
Color	MBL2/JAK2/TLR4 genes	PCR-PEXT	Antibiotin–AuNPs	-	**5 SNPs** **10 alleles**	[73]
Color	Plant edible oils	PCR-PEXT	Antibiotin–AuNPs	-	**7**	[74]
Color	Olive oil	PCR-PEXT	Antibiotin–AuNPs	-	2 SNPs **4 alleles**	[75]
***LAMP***						
Color	SARS-CoV-2 virus	LAMP	SA–polymer NPs	12 copies	2	[76]
Color	*Salmonella* spp./*Cronobacter* spp.	LAMP	Anti-FITC Ab–AuNPs	2.8–4.3 CFU/g	2	[77]
Color	HPV virus	LAMP	Antibiotin–AuNPs	10–100 copies/reaction, 1 ng/reaction	2	[78]
Color	*Raillietina* spp./*Ascaridia galli* bacteria	LAMP	Anti-FAM Ab–AuNPs	5 pg/μL	2	[79]
Color	*Salmonella/Shigella* bacteria	LAMP	SA–AuNPs	3.4–3.9 CFU/reaction	2	[80]
Color	*Salmonella*/*Staphylococcus* bacteria	LAMP	Antibiotin–AuNPs	16 CFU, 9 aM, 5.4 copies/mL	2	[81]
***RPA***						
Color	Bovine viral diarrhea virus/bovine epidemic fever virus/bovine respiratory syncytial virus	RT-RPA	Antibiotin–AuNPs	262, 242, and 256 copies/μL	**3**	[82]
Color	SARS-CoV-2 virus/MS2 Bacteriophage	RT-RPA	Anti-FAM Ab–AuNPs	50 copies	2	[83]
Color	*Staphylococcus aureus/Vibrio parahaemolyticus/Salmonella Enteritidis*	RPA	Anti-Dig Ab–AuNPs	12.9–76 CFU/mL	**3**	[84]
Color	African swine fever virus	RPA/CRISPR	Anti-FAM Ab–AuNPs	1.6 and 8 copies/μL	2	[85]
Color	Cauliflower Mosaic Virus 35S promoter/nopaline synthase terminator, color terminator	RPA/CRISPR	Anti-FITC/Dig Ab–AuNPs	20 copies	2	[86]
Color	HPV virus	RPA/CRISPR	Anti-FAM/Dig Ab–AuNPs	10 copies/μL	2	[87]
Color	GM crops	RPA-CRISPR/Cas12a	Anti-FAM/Dig Ab–red or blue latex microspheres	10 copies/μL	2	[88]
Color	Meat species Mitochondrial gene *Anas platyrhynchos* for duck/*Gallus gallus* for chicken	RPA	DNA–magenta AuNPs/cyan gold nanoflowers	0.01%	2	[89]
Color	Meat species Pork (*Porcine*) mitochondrial ND2 gene/chicken (*Gallus gallus*) cytochrome B gene	RPA	SA–AuNPs	0.1%	2	[90]
Color	Tobacco potyviruses	RT-RPA	Antibiotin–AuNPs	10^3^ copies	**3**	[91]
***Other***						
Color	miR-210/miR-424	Hybridization	DNA–AuNPs	10 pmol	2	[92]
Color	miR-21/miR-155/miR-210	Hybridization	DNA–AuNPs	0.007 nM, 0.068 nM, and 0.017 nM	**3**	[4]
Color	H1N1/H3N2/ H9N2 influenza virus synthetic nucleic acids sequences	Hybridization	DNA–Fe_3_O_4_–Au NPs	0.5–2.5 nM	**3**	[93]
Fluorescence	Bacterial 16S rRNA *Staphylococcus aureus/Listeria* *Monocytogenes/Salmonella typhimurium*	HCR/Exo-III amplifier	DNA–CdTe/CdS	47, 43, and 56 CFU/mL	**3**	[94]
SERS	miR-106b/miR-196b	SERS/CHA	DNA–Pd–Au nanorods–hairpin	43.08 and 61.36 aM	2	[95]
SERS	Horse meat adulteration	Hybridization/SERS/RPA	AuNPs–DNA probes	0.01%	2	[96]
***Aptamer***						
Fluorescence	Hg^2+^/ochratoxin A/*Salmonella*	Competitive aptamer–LFA	DNA–UCNPs	5 ppb, 3 ng/mL, and 85 CFU/mL	**3**	[55]
Color	Aflatoxin M1/aflatoxin B1/ochratoxin A	Aptamer–LFA/hybridization	DNA–Au@Ir NPs	0.39 ng/mL, 0.36 ng/mL, and 0.82 ng/mL	**3**	[97]
**Combined biomolecules categories**						
Fluorescence	*Pseudomonas auruginosa* gDNA/IL-6 protein	RPA and sandwich immunoassay	Microspheres	70 copies/reaction gDNA and 4 ng/mL protein	2	[99]

**Ab**, antibody; **AuNPs**, gold nanoparticles; **Au@Ir NPs**, gold-iridium nanoparticles; **CDs**, carbon dots; **CHA**, catalytic hairpin assembly; **CRISPR**, clustered regularly interspaced short palindromic repeats; **Dig**, digoxigenin; **DMSNs-BCDs**, phosphorus-doped carbon-dot-based dendritic mesoporous silica nanoparticles; **EuNPs**, europium nanoparticles; **Exo**, exonuclease; **FAM**, fluorescein amidite; **FITC**, fluorescein isothiocyanate; **gDNA**, genomic DNA; **HCR**, hybridization chain reaction; **LAMP**, loop-mediated isothermal amplification; **LFA**, lateral flow assay; **NPs**, nanoparticles; **PCR**, polymerase chain reaction; **PEXT**, primer extension reaction; **QDs**, quantum dots; **RPA**, recombinase polymerase isothermal amplification; **RT**, reverse transcription; SA, streptavidin; **TRFMs**, time-resolved microspheres; **UCNPs**, upconversion nanoparticles.
